# Recent advances in the role of polysaccharides in liver diseases: a review

**DOI:** 10.3389/fphar.2025.1535717

**Published:** 2025-03-27

**Authors:** Junfeng Wang, Hanxiang Wang, Xiawen Yang, Kaiping Wang, Yu Zhang

**Affiliations:** ^1^ Department of Pharmacy, Union Hospital, Tongji Medical College, Huazhong University of Science and Technology, Wuhan, China; ^2^ Hubei Province Clinical Research Center for Precision Medicine for Critical Illness, Tongji Medical College, Huazhong University of Science and Technology, Wuhan, China; ^3^ Department of Pharmacy, The Central Hospital of Wuhan, Tongji Medical College, Huazhong University of Science and Technology, Wuhan, China; ^4^ Hubei Key Laboratory of Nature Medicinal Chemistry and Resource Evaluation, Tongji Medical College of Pharmacy, Huazhong University of Science and Technology, Wuhan, China

**Keywords:** polysaccharides, hepatoprotective effects, liver diseases, drug carrier, mechanism

## Abstract

Liver diseases are a serious health problem worldwide, especially with a sustained increase in the burden of it every year. However, drugs commonly used in patients have limited efficacy and serious adverse reactions associated with long-term use. Therefore, it is urgent to find effective and safe alternatives. Polysaccharides are currently considered promising alternatives to traditional drugs because of their extensive activity and low toxicity. This review investigated the studies on hepatoprotective polysaccharides over the past 6 years, detailing their hepatoprotective effects, potential mechanisms, and drug carrier applications. These findings suggest that polysaccharides have prominent preventive and therapeutic effects on various liver diseases such as drug-induced liver injury, alcoholic liver disease, hepatitis B, non-alcoholic fatty liver disease, liver fibrosis, and hepatocellular carcinoma. Its mechanism includes multiple aspects such as metabolic regulation, reduction of oxidative stress and inflammation, and regulation of gut microbiota. Furthermore, owing to the good physicochemical properties, polysaccharides have been applied in delivery systems for chemotherapy drugs and small molecule drugs. However, further research is essential on the bioavailability, structure-activity relationship, and more clinical evidence of polysaccharides. Continued exploration of polysaccharides will provide tremendous potential for the treatment of liver diseases.

## 1 Introduction

**FIGURE 1:**
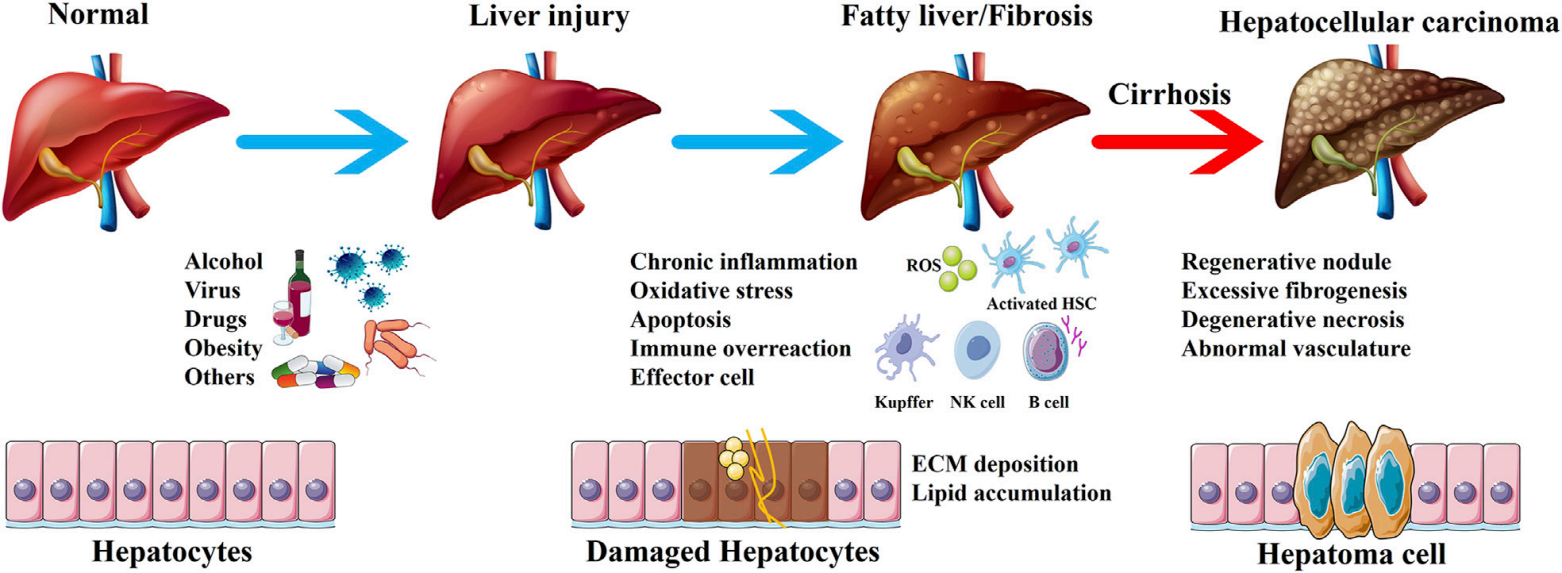
The development of liver diseases. The liver is exposed to pathogenic factors, and chronic liver injury leads to inflammation, oxidative stress, apoptosis and immune response. Activated effector cells promote fatty liver and liver fibrosis, ultimately resulting in cirrhosis and hepatocellular carcinoma.

The liver is mainly responsible for metabolism and detoxification in the body. The functional characteristics of the liver make it vulnerable to damage from various exogenous substances such as viruses, drugs, alcohol, and chemicals (Pei et al., 2023). Studies have shown that alcoholism, non-alcoholic steatohepatitis, and viral infections are the three major inducement of liver injury (Pimpin et al., 2018). Persistent liver injury can disrupt the normal structure and function of the liver, leading to fatty liver, fibrosis, and even cirrhosis. The mechanism of occurrence and development of liver diseases is complex. Based on the available literature, the concise progress involving the development of liver diseases has been summarized (Figure 1). Liver diseases account for approximately 2 million deaths annually, which represents 4% of all global deaths (Devarbhavi et al., 2023). Cirrhosis and liver cancer, the terminal stages of liver diseases, are the predominant causes of death, with a persistently poor 5-year survival rate for patients undergoing liver transplantation (Terrault et al., 2023). Thus, it seems to be of great significance to focus on prevention and treatment strategies in the early stages of liver diseases. Currently, the treatment of liver diseases in the early stage is mainly based on drugs such as silymarin and the derivatives of glycyrrhizic acid. However, the long-term use of existing drugs increases the incidence of adverse events and may even induce further drug-induced liver injury. Therefore, it is crucial to identify new hepatoprotective drugs or adjuvants with low toxicity and high efficiency.

In recent years, drugs from natural sources have become increasingly favored in medicine and healthcare. Polysaccharides are important active ingredients that are widely present in medicinal plants and fungi such as *Ganoderma lucidum*, *Astragalus membranaceus*, and *Angelica sinensis* (Yuan et al., 2019). Polysaccharides have attracted increasing attention in ethnopharmacology due to their low toxicity and extensive pharmacological activities, such as antioxidant, anti-inflammatory, immunomodulatory and anti-tumor (Guo et al., 2023). Studies have found that polysaccharides have protective effects on liver diseases through various mechanisms both *in vivo* and *in vitro* (Yuan et al., 2019). In addition, polysaccharides are rich in active functional groups, such as carboxyl, hydroxyl, and amino groups, which make them easy to modify. This facilitates the application of polysaccharides as drug carriers for delivering hepatoprotective drugs, suggesting the potential of polysaccharides in the field of biomaterials (Barclay et al., 2019).

Some reviews investigated the effects of plant polysaccharides on several liver diseases and the signaling pathways through which plant polysaccharides regulate inflammation, apoptosis and oxidative stress (Qu H. et al., 2020; Wei et al., 2024). These previous reviews provided a foundation for the management of liver diseases by plant polysaccharides. However, the overall understanding of the hepatoprotective effects and applications of polysaccharides is still insufficient, which impedes the further development of polysaccharides in the field of liver protection. Due to the rapid increase in the quantity of bioactive polysaccharides, the mechanisms and functionalities of polysaccharides with hepatoprotective effects discovered in recent years have yet to be systematically summarized. In this review, the hepatoprotective effects and underlying mechanisms of polysaccharides from plants and fungi in drug-induced liver injury, alcoholic liver disease, hepatitis B, non-alcoholic fatty liver disease, liver fibrosis and hepatocellular carcinoma are summarized, primarily covering the researches from January 2019 to October 2024. Moreover, the application of polysaccharides as drug carriers in liver diseases has also been summarized, which is the emerging functional research of polysaccharides against liver diseases. This review intends to provide theoretical basis for hepaprotective effects of polysaccharides and new treatment strategies for liver diseases.

## 2 Methods

### 2.1 Search strategy

Primary studies from January 2019 to October 2024 were searched in major online databases (Web of Science, PubMed, and CNKI). The keywords used in the search included polysaccharide, liver disease, drug-induced liver injury, alcoholic liver disease, non-alcoholic fatty liver disease, hepatitis B, liver fibrosis, hepatocellular carcinoma, and drug carriers, as well as a combination of these words. These studies were systematically classified according to their main objectives, and their major findings were summarized for clarity. The original documents, which are unrelated to the subject, non-pharmacological, repetitive and inaccessible, are excluded.

### 2.2 Critical review of literature

The documents were critically reviewed according to guidelines (Heinrich et al., 2020). Specifically, the models, controls, and methodological details were taken into consideration. Tables 1–6 includes the pharmacological documents after assessment, and the original data are shown in Supplementary Table S1.

**TABLE 1:** Polysaccharides against DILI.

Polysaccharide	Source	Model	Mechanism	References
*Smilax china* L. polysaccharide	*Smilax china* L.	APAP-induced BALB/c	Upregulated antioxidant levels and activated Nrf2-ARE pathway	Wang M. et al. (2022)
*Schisandra chinensis* acidic polysaccharide	*Schisandra chinensis* (Turcz.) baill	APAP-induced ICR	Decreased MDA and GSH depletion, and elevated expression of Nrf2 and HO-1	Che et al. (2019)
*Phellinus linteus* polysaccharide	*Phellinus linteus*	APAP-induced Nrf2^−/−^	Activated AMPK/Nrf2 pathway and antioxidant enzymes	Zhao Y. et al. (2022)
Polysaccharides of *Polygonum multiflorum* Thunb in raw and processed products	*Polygonum multiflorum* Thunb	APAP-induced Kunming	Upregulated antioxidant enzyme, repressed lipid peroxidation	Wang Y. et al. (2023)
*Phellinus linteus* polysaccharide	*Phellinus linteus*	APAP-induced Kunming	Reduced levels of CYP2E1 and oxidative stress, and accelerated the metabolism of APAP	Chen et al. (2020)
*Prunella vulgaris* sulfated polysaccharide	*Prunella vulgaris* L.	INH-induced C57BL/6J	Improved SOD activity, reduced the levels of IL-6 and TNF-α	Zhuang et al. (2022)
*Echinacea purpurea* polysaccharide	*Echinacea purpurea* (L.) Moench	APAP-induced C57BL/6J	Increased autophagy by Parkin-dependent pathway	Yu et al. (2022)

**TABLE 6:** Polysaccharides against HCC.

Polysaccharide	Source	Model	Mechanism	References
*Ganoderma lucidum* polysaccharide	*Ganoderma lucidum* (Curtis) P. Karst.	Hepa1-6-bearing C57BL/6	Modulated the polarization of macrophage via MAPK/NF-κB pathway	Li M. et al. (2023)
Basil polysaccharide	*Ocimum basilicum* L.	MHCC97H-bearing nude mice	Inhibited the metastasis of tumor via HIF-1α-mediated epithelial-mesenchymal transition	Feng et al. (2019)
*Asparagus* polysaccharide	*Asparagus officinalis* L.	SK-Hep1、Hep-3B cells	Inhibited the invasion, migration and angiogenesis of HCC cells via HIF-1*α*/VEGF pathway	Cheng et al. (2019)
*Grifola frondose* polysaccharide	*Grifola frondosa* (Dicks.) Gray	H22, HepG2 cells	Induced the caspases-dependent apoptosis of HCC cells through mitochondria apoptotic pathway	Yu et al. (2020)
Mulberry fruit polysaccharide	*Murus alba* L.	DEN/PB induced-HCC in rat	Induced apoptosis by regulating apoptosis proteins (Bcl-2, Bax, caspase3 and caspase9) and reduced the inflammation markers	Li et al. (2021)
*Pleurotus ostreatus* polysaccharide	*Pleurotus ostreatus*	H22 bearing-BALB/c	Increased apoptosis in HCC cells by regulating cytochrome c	Khinsar et al. (2021)
*Rhizopus nigricans* polysaccharide	*Rhizopus nigrum*	HepG2-bearing nude mice	Inhibited the proliferation of HCC cells and the growth of tumor via miR-494-3p/TRIM36 axis	Yan et al. (2022)
Undeproteinized *Flos Sophorae Immaturus* polysaccharide	*Sophora japonica* L.	SMMC 7721 cells	Inhibited the migration of SMMC 7721 cells and promoted apoptosis	Zhong et al. (2022)
Papain-deproteinized *Flos Sophorae Immaturus* polysaccharide				
*Dandelion* polysaccharide	*Taraxaci Herba*	Hepa1-6 or H22-bearing BALB/c	Decreased iron burden by regulating the expression of hepcidin via JAK/STAT pathway	Ren et al. (2021)
*Astragalus* polysaccharide	*Astragalus membranaceus* (Fisch.) Bge.	Hep3B bearing-BALB/c	Increased doxorubicin-induced apoptosis by reducing the O-GlcNAcylation	Li G. L. et al. (2023)
Neutral *Panax notoginseng* polysaccharide	*Panax notoginseng* (Burk.) F.H.Chen	H22 bearing-Kunming	Increased the tumor inhibition rate of cyclophosphamide by enhancing immunity	Liu X. et al. (2021)

### 2.3 Terminology

To ensure the accuracy and clarity of terminology, we standardized the terms of plant and fungal parts according to previous research as follows (Lei et al., 2021): Tuberous root (tuber, rhizome), Fibrous root (fibrous root), Fruit (Fruit, seed), Aerial part (leaf, flower), Mycelia (Mycelia, fruiting body), Sclerotium (sclerotium).

## 3 The effects of polysaccharides in liver diseases

### 3.1 Drug-induced liver injury (DILI)

The liver, as the primary organ for drug metabolism, is susceptible to damage caused by medications. DILI, one of the main reasons of liver diseases worldwide, is the acute or chronic liver injury triggered by abnormal liver function as the result of drugs and its metabolites (Bjornsson and Bjornsson, 2022; Yu C. et al., 2023). Acetaminophen (APAP), isoniazid, and valproic acid are common examples of DILI, resulting in acute liver failure with potentially fatal consequences. The advancement of strategies to prevent DILI not only holds great significance for liver injury, but may also enhance the availability of numerous medications used to a wide range of diseases. Many polysaccharides have shown therapeutic effects on DILI (Table 1).

Drugs or their metabolites reduce antioxidant capacity by disrupting mitochondrial function, and the accumulation of reactive oxygen species (ROS) damages deoxyribonucleic acid (DNA) and proteins in liver cells, ultimately leading to cell death (Ye et al., 2018). Therefore, the improvement of liver antioxidant system is beneficial for DILI. Nuclear factor erythroid 2-associated factor 2/antioxidant response element (Nrf2/ARE) pathway is the major regulatory pathway against oxidative stress of liver injury (Zhou et al., 2022). *Smilax china* L. polysaccharide, *Schisandra chinensis* acidic polysaccharide and *Phellinus linteus* polysaccharide upregulated antioxidant capacity through Nrf2 pathway, exerting therapeutic effects on DILI (Che et al., 2019; Wang K. et al., 2022; Zhao H. et al., 2022). Both polysaccharides of *Polygonum multiflorum* Thunb in raw and processed products upregulated antioxidant enzymes and repressed lipid peroxidation in APAP-induced liver injury (Wang D. et al., 2023). Cytochrome P450 proteins (CYP), the key enzyme in drug metabolism, plays an important role in liver injury. Several subtypes such as CYP2E1 and CYP3A4 could alleviate DILI (Wang K. L. et al., 2019). *Phellinus linteus* polysaccharide reduced expression of CYP2E1 and oxidative stress in the liver of mice with liver injury, and encouraged the metabolism of APAP (Chen et al., 2020). In addition, inhibition of inflammatory mediators can alleviate DILI. *Prunella vulgaris* sulfated polysaccharide protected mice from isoniazid-induced liver injury by reducing inflammatory factor, such as interleukin-6 (IL-6) and tumor necrosis factor α (TNF-α) (Zhuang et al., 2022). Autophagy is a self-degrading mechanism that is crucial for the cell survival and internal environment stability. The liver relies on autophagy to maintain normal function and prevent the development of disease, and changes in autophagy are potential mechanisms underlying many common liver diseases (Qian et al., 2021). The effect of *Echinacea purpurea* polysaccharide against APAP-induced liver injury was attributed to a reduction in autophagy-dependent inflammation and oxidative stress (Yu et al., 2022).

Collectively, polysaccharides demonstrate notable protective effects against DILI, with mechanisms involving antioxidant and anti-inflammatory activities. In general, the important strategy for treating liver injury is to enhance the antioxidant capacity and reduce inflammation in liver. However, the imbalance of bile acid homeostasis has recently become an important factor in DILI, and drug-induced cholestasis can induce hepatocyte damage (Kolaric et al., 2019). Currently, there is limited research on the role of polysaccharides in cholestatic hepatitis. Regulation of bile acid homeostasis may be a new direction for future research on polysaccharides against DILI. In addition, there are no clinical studies on the effects of polysaccharides on DILI. Therefore, a necessary step to promote the development of polysaccharide drugs is to conduct clinical trials as soon as possible.

### 3.2 Alcoholic liver disease (ALD)

Persistent or excessive alcohol consumption mainly accounts for ALD. Statistics indicated the annual mortality of ALD worldwide has exceeded that of hepatitis C virus (Yang K. et al., 2020). Meanwhile, alcoholic hepatitis has become the second motivation of end-stage liver diseases in China, while viral hepatitis ranks first (Wenjun et al., 2019). The effective treatment strategies for ALD are alcohol abstinence and the assistance of hepatoprotective medicines. However, there are some problems that the poor compliance of some patients and side effects in long-term administration of commonly used therapeutic drugs. Therefore, it is crucial to find safe and effective drugs for ALD. Importantly, researches reveal that the evident anti-ALD effect is reflected in polysaccharides, which may be a good choice for drug development (Table 2).

**TABLE 2:** Polysaccharides against ALD.

Polysaccharide	Source	Model	Mechanism	References
*Echinacea purpurea* polysaccharide	*Enteromorpha prolifera*	ALD in C57BL/6	Regulated the activity of CYP2E1, ADH and ALDH, and activated Nrf2/HO-1 pathway	Yan T. et al. (2024)
*Pleurotus geesteranus* polysaccharide	*Pleurotus geesteranus*	ALD in Kunming	Improved liver structure, regulated alcohol metabolism and restored the levels of serum lipid	Song S. et al. (2021)
*Angelica sinensis* polysaccharide	*Angelica sinensis* (Oliv.) Diels	AFLD in Balb/C	Suppressed lipid synthesis and uptake through AMPK-SIRT1 pathway	He L. et al. (2022)
*Polygala fallax* Hemsl polysaccharide	*Polygala fallax* Hemsl	AFLD in C57BL/6	Regulated lipid synthesis and inhibited lipid accumulation via AMPK pathway	Lv et al. (2024)
*Pinus koraiensis* pine nut polysaccharide	*Pinus koraiensis* Sieb. Et Zucc.	ALD in Kunming	Alleviated oxidative stress through Nrf2/HO-1 pathway, inhibited inflammation and promoted the repair of DNA	Qu J. et al. (2020)
*Morchella esculenta* polysaccharide	*Morchella esculenta*	ALD in C57BL/6	Regulated inflammatory cytokines and oxidative stress through Usp10/NF-KB/Nrf2 axis	Teng et al. (2023)
*Coriolus versicolor* polysaccharide	*Coriolus versicolor*	ALD in C57BL/6	Regulated inflammatory cytokines through TLR4 pathway and improved oxidative stress	Wang Y. X. et al. (2019)
*Dendrobium officinale* polysaccharide	*Dendrobium officinale* Kimura et Migo	ALD in SD	Reduced the expression of TNF-α, IL-1β, IL-6 through TLR4/NF-KB signaling pathway	Yang K. et al. (2020)
*Echinacea purpurea* polysaccharide	*Echinacea purpurea* (L.) Moench	ALD in Kunming	Upregulated the activity of antioxidant enzymes and protected intestinal barrier	Jiang et al. (2021)
*Poria cocos* polysaccharide	*Poria cocos* (Schw.) Wolf	ALD in C57BL/6	Reduced oxidative stress and inflammation, and improved intestinal barrier injury	Jiang Y. H. et al. (2022)
Alhagi honey polysaccharide	*Alhagi sparsifolia* Shap	ALD in C57BL/6	Protected intestinal barrier, eliminated oxidative stress and inhibited inflammation	Song et al. (2024)
*Schisandra chinensis* polysaccharide	*Schisandra chinensis* (Turcz.) baill	ALD in C57BL/6	Modulated the gut microbiota and tryptophan metabolism	Chi et al. (2024)
*Nostoc commune* Vauch. Polysaccharide	*Nostoc commune* Vauch.	ALD in Kunming	Improved gut microbiota disturbance and antioxidant capacity	Yang et al. (2023)

The pathogenesis of ALD is complicated, which may involve the disturbance of alcohol or lipid metabolism, oxidative stress, the influence of inflammatory factors, and the homeostasis of intestinal flora (Chen C. et al., 2023; Wu et al., 2023). Alcohol is mainly metabolized in the liver through the alcohol dehydrogenase (ADH) system. Usually, excessive alcohol consumption can reduce ADH activity and increase CYP2E1 activity, leading to alcohol metabolism disorders (Jiang et al., 2020). Presently, there have been many reports on the activation of liver ADH by polysaccharides. Both of *Enteromorpha prolifera* polysaccharide and *Pleurotus geesteranus* polysaccharide enhanced alcohol metabolism by increasing ADH activity and reducing CYP2E1 activity in mice with ALD (Song S. et al., 2021; Yan T. et al., 2024). In addition, alcohol can induce lipid metabolism disorders in the liver, leading to fat accumulation and the formation of fatty liver (You and Arteel, 2019). Research has shown that AMP-activated protein kinase (AMPK) pathway regulates lipid metabolism (Fang et al., 2022). *Angelica sinensis* polysaccharide could significantly inhibit lipid synthesis and uptake via AMPK/sirtuin 1 (SIRT1) pathway (He Z. et al., 2022). *Polygala fallax* Hemsl polysaccharide alleviated alcoholic fatty liver disease by regulating lipid metabolism through AMPK pathway (Lv et al., 2024). The overproduction of ROS resulting from excessive alcohol intake could disrupt the redox balance, and oxidative stress may be an important factor in ALD (Chen M. et al., 2023). The impact of polysaccharides on the antioxidant defense system has been extensively studied in ALD models. Polysaccharides from *Pinus koraiensis* Sieb. et Zucc. and *Morchella esculenta* alleviated liver injury in alcohol-induced mice by inhibiting oxidative stress via the Nrf2 pathway (Qu J. et al., 2020; Teng et al., 2023). Alcohol-induced inflammatory factors are the main cause of immune imbalance, which can exacerbate liver damage (Hosseini et al., 2019). The anti-inflammatory effect of polysaccharides has received extensive attention as an important mechanism of liver protection. *Coriolus versicolor* polysaccharide ameliorated alcohol-induced liver injury by modulating inflammatory cytokines through toll-like receptor 4 (TLR4) pathway (Wang Y. X. et al., 2019). Similarly, *Dendrobium officinale* polysaccharide protected against ethanol-induced acute liver injury *in vivo* and *in vitro* by reversing the expression of TNF-α, interleukin-1β (IL-1β) and IL-6 through TLR4/nuclear factor-kappa B (NF-κB) pathway (Yang Y. et al., 2020). In addition, the homeostasis of gut microbiota is closely related to the body’s immune, metabolic, and other biological functions. Excessive alcohol consumption may disrupt the intestinal barrier and microbial homeostasis (Arab et al., 2018). Recently, polysaccharides have been reported to regulate gut microbiota through the gut liver axis to prevent and treat ALD. *Echinacea purpurea* polysaccharide, *Poria cocos* polysaccharide, and Alhagi honey polysaccharide have been shown therapeutic effects in ALD mice by protecting the intestinal barrier (Jiang et al., 2021; Jiang Y. H. et al., 2022; Song et al., 2024). *Schisandra chinensis* polysaccharide prevented ALD by enriching intestinal *Lactobacillus* and regulating tryptophan metabolism (Chi et al., 2024). *Nostoc commune* Vauch. polysaccharide improved gut microbiota composition by increasing beneficial bacteria and reducing harmful bacteria in acute ALD mice (Yang et al., 2023).

In summary, polysaccharides derived from medicinal plants and fungi exhibit excellent preventive and protective effects on ALD, serving as potential drugs against it and possessing great development value. The mechanisms by which polysaccharides alleviate ALD include activation of enzymes related to alcohol metabolism, reduction of oxidative stress, inhibition of inflammation, improvement of lipid metabolism, and regulation of gut microbiota. Importantly, alcohol metabolism involves multiple organs, including the stomach, intestines, and liver, and the first-pass metabolism of alcohol in the gastric mucosa alleviates the toxicity of alcohol through the reduction of its bioavailability. Therefore, future research on the role of polysaccharides in alcohol metabolism can also focus on the absorption capacity of the stomach. Moreover, there are few studies on the role of functional groups in the activities of polysaccharides, which hinders the development of polysaccharides as innovative drugs or food additives. Further analysis of the structure-activity relationship of polysaccharides is of great significance in the treatment of ALD.

### 3.3 Hepatitis B

Viral hepatitis characterized by viral infection is a globally recognized health issue. Although the widespread use of hepatitis B virus (HBV) vaccine has significantly diminished the infection rate of HBV, more than 250 million individuals chronically infected with HBV in the world (Prange, 2022). Hepatitis B and its complications accounts for more than 880 thousand deaths every year, with a high risk of developing liver cirrhosis and cancer (Herrscher et al., 2020). To date, the strategies for HBV remains problem from low cure rates and side effects. However, the role of purified natural products such as polysaccharides in the prevention and treatment of viral infection cannot be overestimated. A variety of polysaccharides with anti-HBV activity are shown in Table 3.

**TABLE 3:** Polysaccharides against HBV.

Polysaccharide	Source	Model	Mechanism	References
*Radix Isatidis* polysaccharide	*Isatis indigotica* Fort.	HepG2.2.15	Reduced the levels of HBV antigens and inhibited DNA replication by JAK/STAT pathway	Wang et al. (2020b)
*Viscum coloratum* (Kom.) Nakai polysaccharide	*Viscum coloratum* (Kom.) Nakai	HepG2.2.15	Decreased the secretion of HBV antigens and the replication of DNA in a dose-dependent manner	Chai et al. (2019)
Flaxseed heteropolysaccharide	*Linum usitatissimum* L.	HepG2.2.15	Decreased the levels of HBeAg, HBsAg and the replication of DNA, and increased mRNA expression of inflammatory cytokines	Liang et al. (2019)
*Saussurea laniceps* polysaccharide	*Saussurea laniceps *Hand.-Mazz.	HepG2.2.15	Reduced the secretion of HBsAg and HBeAg	Chen et al. (2019)
*Strongylocentrotus nudus egg* polysaccharide	*Strongylocentrotus nudus*	HBV-infected C57BL/6	Reduced the secretion of HBV antigens, and inhibited the replication of HBV through TLR4-induced immune pathway	Yu H. et al. (2023)

Hepatitis B surface antigen (HBsAg) is the most important serological markers for the diagnosis of HBV infection, and may contribute to liver injury by HBV infection (Chuang et al., 2022). Thus, inhibition of HBsAg is a reliable indicator for screening anti-HBV drugs. Polysaccharides extracted from *Isatis indigotica* Fort (RIP), *Viscum coloratum* (Kom.) Nakai, *Linum usitatissimum* L (FP-1), *Saussurea laniceps* Hand.-Mazz. and *Strongylocentrotus nudus* (SEP) have significant inhibitory effects on HBsAg (Chai et al., 2019; Chen et al., 2019; Liang et al., 2019; Wang et al., 2020a; Yu H. et al., 2023). It is worth noting that most studies have demonstrated that polysaccharides can inhibit the production and secretion of HBsAg *in vitro*, and there is a lack of experimental verification and deeper mechanism research *in vivo*. Increasing evidence has shown that polysaccharides could inhibit virus replication via immune system. RIP efficiently inhibited HBV-DNA replication through activation of interferon α-dependent janus kinase/signal transducer and activator of transcription (JAK/STAT) pathway (Wang et al., 2020b). FP-1 showed pro-inflammatory and interference of HBV-DNA replication effects (Liang et al., 2019). Recently, TLR agonists, as immunomodulators, have attracted attention for their potential anti-HBV effects (Lucifora et al., 2018). SEP reduced HBV replication through the activation of TLR4/NF-κB-dependent immune pathway. However, TLR inhibitor did not completely counteract the anti-HBV activity of SEP (Yu X. et al., 2023). Thus, immune regulation is a direction of research on the mechanism of polysaccharides against HBV, and there may be other targets that need to be further explored.

The above studies indicate that polysaccharides exhibit a significant antiviral effect, primarily by inhibiting hepatitis B antigen and interfering with DNA replication. However, studies on polysaccharides against HBV are not yet widespread. Furthermore, most studies have only been conducted at the cellular level, and animal or human studies are greatly needed. The immunomodulatory activity of polysaccharides is beneficial for antiviral effects, but the specific mechanisms are still not fully understood. Additionally, polysaccharides have been proven to possess characteristics such as intrinsic immunomodulation, biocompatibility, low toxicity, and safety when used as vaccine adjuvants (Sun et al., 2018). Therefore, it is speculated that exploring polysaccharides as vaccine adjuvants to enhance immunity could become a new approach for polysaccharide-based treatment against HBV.

### 3.4 Non-alcoholic fatty liver disease (NAFLD)

NAFLD is the most prevailing chronic liver diseases worldwide, with a global prevalence of more than 25% (Guo et al., 2023). The characteristic of NAFLD is the confusion of lipid metabolism induced by non-alcoholic hepatic steatosis, which may lead to liver cirrhosis and even cancer. To date, the effective strategy for NAFLD is the reduction of at least 5% of body weight in obese patients, and there are no specific medicines approved for the treatment of NAFLD (Ferro et al., 2020). Recently, the regulatory effect of polysaccharides on metabolic processes and intestinal microbiota has attracted widespread attention, and there is increasing evidence that polysaccharides inhibit the occurrence and development of NAFLD. As shown in Table 4, the polysaccharides against NAFLD are collected.

**TABLE 4:** Polysaccharides against NAFLD.

Polysaccharide	Source	Model	Mechanism	References
*Angelica sinensis* polysaccharide	*Angelica sinensis* (Oliv.) Diels	HFD-induced BALB/c	Increased the level of propionate and regulated lipid metabolism	Luo et al. (2023)
*Astragalus* polysaccharide	*Astragalus membranaceus* (Fisch.) bunge.	HFD-induced C57BL/6	Increased the levels of taurohyodeoxycholic acid, regulated lipid metabolism enzymes	Zheng et al. (2024)
*Chaetomorpha linum* polysaccharide	*Chaetomorpha linum*	HFD-induced C57BL/6J	Limited body weights and liver lipid deposition by PPARα/CPT-1/MCAD pathway	Chu et al. (2022)
*Oudemansiella raphanipies* polysaccharide	*Oudemansiella raphanipies*	HFD-induced C57BL/6J	Modulated liver metabolic profiling, and decreased lipid droplet accumulation	Jiang H. et al. (2022)
*Lycium barbarum* polysaccharide	*Lycium barbarum* L.	HFD-induced SD	Regulated intestinal microbiota balance, restored intestinal barrier and inhibited inflammation	Gao et al. (2021a)
*Ophiopogon japonicus* polysaccharide	*Ophiopogon japonicus*	HFD-induced C57BL/6J	Regulated lipid-related pathway via modulating the abundance of *Akkermansia miniciphila*	Zhang L. et al. (2022)
*Coriolus versicolor* polysaccharide	*Coriolus versicolor*	HFD-induced C57BL/6J	Reduced intestinal microbiota, especially bile acids-related microbes	Tang et al. (2023)
*Poria cocos* polysaccharide	*Poria cocos* (Schw.) Wolf	HFD-induced C57BL/6J	Improved the gut-vascular barrier, inhibited the endotoxin translocation and pyroptosis of macrophages	Ye et al. (2022)
*Salviae miltiorrhizae* polysaccharide	*Salviae miltiorrhizae* Bunge	HFD-induced C57BL/6	Modulated the homeostasis of gut microbiota and improved intestinal function	Li L. et al. (2022)
*Polygonatum cyrtonema* Hua polysaccharide	*Polygonatum cyrtonema* Hua	HFD-induced C57BL/6J	Improved lipid metabolism, oxidative stress and intestinal microbiota	Liu et al. (2022)
*Crataegus pinnatifida* polysaccharide	*Crataegus pinnatifida* Bunge	HFD-induced C57BL/6J	Regulated intestinal microbiota imbalance and the production of short-chain fatty acid	Hao et al. (2024)
*Atractylodes macrocephala* Koidz polysaccharide	*Atractylodes macrocephala* Koidz	HFD-induced C57BL/6	Alleviated hepatic inflammation through regulating the TLR4/MyD88/NF-κB pathway	Chen et al. (2024)
*Rosa chinensis* polysaccharide	*Rosa chinensis* Jacq.	CCl_4_-induced C57BL/6J	Inhibited inflammation through HMGB1/TLR4/NF-κB pathway	Jing et al. (2024)
*Codonopsis pilosula* polysaccharide	*Codonopsis pilosula* (Franch.) Nannf.	HFD-induced C57BL/6	Increased the levels of SOD and CAT, and reduced MDA	Ma et al. (2024)
*Sagittaria sagittifolia* polysaccharide	*Sagittaria sagittifolia* L.	MCD diet-induced ICR	Interfered the metabolism of arachidonic acid through Nrf2/HO-1 pathway	Deng et al. (2020)
*Stropharia rugoso-annulata* acetylated polysaccharide	*Stropharia rugoso-annulata*	HFD-induced Kunming	Modulated oxidative stress and lipid metabolism through Nrf2/JNK1/AMPK axis	Li X. et al. (2022)
Seabuckthorn polysaccharide	*Hippophae rhamnoides* L.	HFD-induced Wistar rats	Mitigated hepatic oxidative stress and steatosis by Nrf2/HO-1 pathway	Yan Y. et al. (2024)

The pathogenesis of NAFLD is a complex multifactorial process. Compared to the “two-hit” hypothesis, the “multi-hit” hypothesis, which considers the intricate crosstalk between multiple organs, seems more reasonable for comprehending NAFLD in the present day (Fang et al., 2018). That is, the development of NAFLD involves multiple mechanisms such as abnormal lipid metabolism, oxidative stress, inflammation, gut microbiota and insulin resistance. Lipid accumulation is a major contributor to the progression of NAFLD, and the key approach to treating NAFLD is the restoration of liver lipid metabolism balance. *Angelica sinensis* polysaccharide regulated liver lipid metabolism through the propionic acid/estrogen-related receptor α pathway, ultimately alleviating NAFLD (Luo et al., 2023). *Astragalus* polysaccharide inhibited lipid accumulation by regulating lipid metabolism enzymes (CYP7A1 and CYP7B1) and increasing taurohyodeoxycholic acid (Zheng et al., 2024). *Chaetomorpha linum* polysaccharide alleviated lipid deposition and reduced the gain in body weights in NAFLD mice by enhancing the peroxisome proliferator-activated receptor α/carnitine palmitoyltransferase-1/medium-chain acyl-CoA dehydrogenase (PPARα/CPT-1/MCAD) pathway (Chu et al., 2022). These studies indicate that polysaccharides can effectively improve the lipid metabolism balance in NAFLD by regulating lipid metabolism related enzymes and pathways. Moreover, metabolomics revealed that *Oudemansiella raphanipies* polysaccharide effectively improved lipid metabolism disorders in NAFLD mice. This beneficial effect was attributed to the regulation of liver metabolic profiling (Jiang H. et al., 2022). When the imbalance of gut microbiota occurs, the injuries of intestinal mucosal barriers and the increase of harmful metabolites promotes the progression of NAFLD (Sanna et al., 2019; Lang and Schnabl, 2020). It is of great significance for maintaining a healthy gut microbiota in the interaction between polysaccharides and gut microbiota (Hu et al., 2023). Polysaccharides reshape the composition of gut microbiota in NAFLD, reducing the abundance of pernicious species. The protective function of *Lycium barbarum* polysaccharide (LPB) in NAFLD was provided by an increase of some *Bacteroidetes* and a reduction of *Proteobacteria*, in which *Proteobacteria* were considered an important source of lipopolysaccharide (LPS). This may explain the inhibition of the LPS/TLR4/NF-κB by LPB. Moreover, LPB led to the upregulation of occludin and zona occludens 1 (ZO-1) protein levels, maintaining the intestinal barrier (Gao et al., 2021a). Importantly, clinical research related to LPB is currently underway and results are expected in the near future (Gao et al., 2021b). *Ophiopogon japonicus* polysaccharide inhibited the NAFLD process by regulating lipid-related pathway via modulating the diversity of intestinal microbiota, especially *Akkermansia miniciphila* (Zhang L. et al., 2022). In addition, polysaccharides from *C. versicolor*, *P. cocos* (Schw.) Wolf, *Salviae miltiorrhizae* Bunge, *Polygonatum cyrtonema* Hua and *Crataegus pinnatifida* Bunge possess beneficial effects on NAFLD mice through regulating intestinal microbiota and strengthening intestinal barriers (Li X. et al., 2022; Liu et al., 2022; Ye et al., 2022; Tang et al., 2023; Hao et al., 2024). Inflammation is considered an important factor in the development of NAFLD, and the investigation has shown that there is a significant correlation between inflammatory cytokines and NAFLD (Duan et al., 2022). Polysaccharides from *Atractylodes macrocephala* Koidz and *Rosa chinensis* Jacq. reduced the NAFLD-induced inflammation through TLR4 pathway (Chen et al., 2024; Jing et al., 2024). The increase of free fatty acids leads to mitochondrial dysfunction, resulting in oxidative stress, which promotes NALFD (Guo et al., 2022). Polysaccharides inhibit the progression of NAFLD through their antioxidant properties. *Codonopsis pilosula* polysaccharide significantly increased the superoxide dismutase (SOD) and catalase (CAT) activities and decreased the malonaldehyde (MDA) activity in NAFLD mice, ameliorating the oxidative stress (Ma et al., 2024). *Sagittaria sagittifolia* polysaccharide regulated arachidonic acid metabolism through the Nrf2/heme oxygenase 1 (HO-1) pathway, reduced oxidative stress, and ultimately alleviated NAFLD (Deng et al., 2020). Similarly, *Stropharia rugoso-annulata* acetylated polysaccharide and Seabuckthorn polysaccharide improved the oxidative stress by Nrf2 pathway, contributing to alleviate the development of NAFLD (Li L. et al., 2022; Yan Y. et al., 2024).

In summary, these findings underscore the complex mechanisms of polysaccharides, suggesting a novel approach for the management of NAFLD. Polysaccharides impede the development of NAFLD by improving lipid metabolism, regulating intestinal flora homeostasis, reducing inflammatory responses, and relieving oxidative stress. Although a small number of clinical studies have been conducted, the majority of research is still limited to rodent models. Therefore, there is an urgent need for large-scale and multicenter clinical trials. It is worth noting that besides effectiveness, the pharmacokinetic and toxicological studies of polysaccharides are also essential for clinical application. In addition, polysaccharides exhibit differential probiotic effects in NAFLD, which may be associated with their structure. The structural characterization of polysaccharides mainly focuses on aspects such as molecular weight, monosaccharide composition, and functional groups, which limits the study of the structure-activity relationship of polysaccharides in NAFLD. Therefore, it is necessary to accurately explore the relationship between polysaccharide structures and specific intestinal flora in order to realize their full potential in NAFLD.

### 3.5 Liver fibrosis

Liver fibrosis is a compensatory response of tissue repair in chronic liver injury, which is characterized by excessive accumulation of extracellular matrix (ECM) components, such as collagen. According to the latest data, liver fibrosis turns into a serious global health problem as it is a necessary stage for chronic liver injury to cirrhosis or liver cancer, and the attention to the prevention and treatment of liver fibrosis is also raised annually (Dhar et al., 2020). Although the understanding of the pathogenesis of liver fibrosis has greatly improved, there is still no effective strategy that can reverse liver fibrosis. Nevertheless, polysaccharides exhibit significant effects against liver fibrosis through various mechanisms, which can be perceived as potential drugs (Wang M. et al., 2022). Polysaccharides against liver fibrosis were collected in Table 5.

**TABLE 5:** Polysaccharides against liver fibrosis.

Polysaccharide	Source	Model	Mechanism	References
*Astragalus* polysaccharide	*Astragalus membranaceus* (Fisch.) Bge.	alcohol-induced SD	Inhibited the co-localisation of TLR4/PTRF and TLR4/JNK/NF-κB pathway	Sun et al. (2023)
*Bletilla striata* polysaccharide	*Bletilla striata* (Thunb.) Reichb. f.	CCl_4_-induced Kunming	Reduced ALT, AST and inflammatory cytokines through TLR2/TLR4-NF-κB pathway	Jiang et al. (2023)
*Dendrobium officinale* polysaccharide	*Dendrobium officinale* Kimura et Migo	CCl_4_-induced SD	Maintained intestinal homeostasis and inhibited inflammation through LPS-TLR4-NF-κB pathway	Wang et al. (2020c)
*Codonopsis pilosula* polysaccharide	*Codonopsis pilosula* (Franch.) Nannf.	CCl_4_-induced Kunming	Decreased collagen deposition and the levels of α-SMA through TLR4/NF-κB and TGF-β1/Smad3 pathway	Meng et al. (2023)
*Ganoderma lucidum* polysaccharide	*Ganoderma lucidum* (Curtis) P. Karst.	CCl_4_-induced C57BL/6	Inhibited inflammation and HSC activation through TLR4/NF-κB and TGF-β/Smad pathway	Chen M. et al. (2023)
*Coprinus comatus* polysaccharide	*Coprinus comatus* (Muell.:Fr.) Gray	CCl_4_-induced C57BL/6	Reduced inflammation and apoptosis by TLR4 and caspase signaling pathway	Zhao L. et al. (2022)
*Aronia melanocarpa* polysaccharide	*Aronia melanocarpa* (Michx.) Elliott	TAA-induced ICR	Reduced ECM production and hepatocyte apoptosis, and modulated gut microbiota	Zhao H. et al. (2022)
*Angelica sinensis* polysaccharide	*Angelica sinensis* (Oliv.) Diels	CCl_4_-induced C57BL/6J	Inhibited activation of HSC via IL-22/STAT3 axis	Wang et al. (2020d)
*Achyranthes bidentata* Bl polysaccharide	*Achyranthes bidentata* Bl	CCl_4_-induced C57BL/6J	Inhibited HSC activation through FAK/PI3K/AKT signaling pathway	Dai et al. (2024)

Inflammatory response is one of the main factors contributing to liver fibrosis. The immune cells recruited after liver injury release inflammatory factors, which activate hepatic stellate cells (HSCs) and promote the development of liver fibrosis (Hammerich and Tacke, 2023). Thus, it is beneficial for alleviating liver fibrosis to reduce inflammation. As is well known, TLR signaling plays a crucial role in chronic liver injury via immune responses mediating inflammation (Khaksari et al., 2024). *Astragalus* polysaccharide improved alcohol-induced liver fibrosis by inhibiting TLR4/c-jun N-terminal kinase (JNK)/NF-κB pathway (Sun et al., 2023). *Bletilla striata* polysaccharide reduced the levels of alanine aminotransferase (ALT) and aspartate aminotransferase (AST), and inhibited the production of inflammatory factors in liver fibrosis through TLRs/NF-κB pathway (Jiang et al., 2023). Moreover, LPS, derived from an imbalanced intestinal environment, exacerbates liver inflammation and promotes liver fibrosis through enterohepatic axis (Zhang et al., 2023). *Dendrobium officinale* polysaccharide alleviated liver fibrosis through intestinal homeostasis and inhibition of inflammatory response mediated by LPS-TLR4-NF-κB pathway (Wang et al., 2020c). The activation of HSCs is the critical event in liver fibrosis and activated HSCs transform into fibroblasts, which secrete collagen and promote the accumulation of ECM. Therefore, inhibition of HSC activation is a key strategy for reversing liver fibrosis. Transforming growth factor β/*drosophila* mothers against decapentaplegic (TGF-β/Smad) signaling pathway plays a vital role in HSC activation, and targeting it may be an important mechanism of polysaccharides in inhibiting the development of liver fibrosis. Polysaccharides from *C. pilosula* (Franch) Nannf. and *G. lucidum* (Curtis) P. Karst. attenuated liver fibrosis via the inhibition of HSC activation mediated by TGF-β/Smad signaling pathway (Chen C. et al., 2023; Meng et al., 2023). Apoptotic bodies generated by hepatocyte apoptosis can be phagocytosed by HSCs, resulting in their activation (New-Aaron et al., 2022). It is also an effective way to prevent hepatocyte apoptosis for inhibiting liver fibrosis. *Coprinus comatus* polysaccharide and *Aronia melanocarpa* polysaccharide ameliorated liver fibrosis through modulating apoptosis (Zhao L. et al., 2022; Zhao Y. et al., 2022). In addition, studies have shown that some signaling axes such as interleukin-22 (IL-22)/STAT3 and focal adhesion kinase/phosphoinositide 3 kinase/protein kinase B (FAK/PI3K/AKT) are involved in the activation process of HSC (Bao et al., 2019; Zhang Y. et al., 2019). *Angelica sinensis* polysaccharide inhibited the activation of HSC by increasing the levels of IL-22 and activating STAT3 pathway in carbon tetrachloride-induced liver fibrosis mice (Wang K. et al., 2020). An inulin-like polysaccharide from *Achyranthes bidentata* Bl inhibited HSC activation through FAK/PI3K/AKT signaling pathway *in vitro* and *in vivo* (Dai et al., 2024).

Collectively, polysaccharides alleviate liver fibrosis via targeting various stages and multiple key pathways in the fibrosis process. Its mechanism is closely related to the regulation of inflammation and the inhibition of HSC activation mediated by TLR4/NF-κB, TGF-β/Smad, and other pathways. However, the study of polysaccharides with anti-hepatic fibrosis properties needs to be improved. There are several new potential targets, such as the Hedgehog pathway and Hippo pathway (Sultana et al., 2025). Mechanisms of anti-fibrotic effects of polysaccharides in this regard have not yet been discovered, which may represent a new direction. Additionally, there are few studies on the structure-activity relationship of polysaccharides with anti-liver fibrosis effects, and the results remain unclear.

### 3.6 Hepatocellular carcinoma (HCC)

HCC, a prevalent malignant tumor, is a grave threat for human health. The morbidity and mortality of HCC remain high, as the second largest cause of cancer related death worldwide (Ince et al., 2020). In recent years, there has been some progress in the treatment of HCC with the development of medicine. However, the serious adverse reactions and drug resistance of targeted small molecule chemotherapy drugs usually influence the therapeutic effect, and the 5-year survival in patients is lower than ideal (He Z. et al., 2022). Therefore, it is urgent to find novel effective drugs for HCC. Many polysaccharides showed significant anti-tumor activity both *in vivo* and *in vitro* (Table 6). Compared to synthetic drugs, polysaccharides without notable side effects are considered as new directions for HCC (Yu et al., 2018; Wang D. et al., 2023).

Immunity is important in contributing to the progression of tumors. Macrophages differentiate into two subtypes, M1 and M2. Interestingly, these two subtypes play different roles in tumor progression, on one hand promoting tumor cell destruction, and on the other hand driving tumor development (Ngambenjawong et al., 2017). Polysaccharides exert anti-tumor effects by regulating immunity. *Ganoderma lucidum* polysaccharide notably inhibited the growth of a Hepa1-6 allograft by modulating the polarization of macrophage via mitogen-activated protein kinase (MAPK)/NF-κB pathway (Li G. L. et al., 2023). Metastasis is the main factors leading to poor prognosis of HCC. Basil polysaccharide inhibited HCC metastasis and progression in xenograft model via hypoxia inducible factor-1*α* (HIF-1α)-mediated epithelial-mesenchymal transition (Feng et al., 2019). As a highly vascularized tumor, the nutrients required for the growth and metastasis of HCC cells are provided by tumor blood vessels, thereby promoting tumor growth. Blocking angiogenesis effectively inhibits tumor progression. Vascular endothelial growth factor (VEGF) is an effective promoter of angiogenesis in tumors (Parmar and Apte, 2021). *Asparagus* polysaccharide reduced migration and angiogenesis of HCC cells through the inhibition of HIF-1α/VEGF signaling pathway (Cheng et al., 2019). Apoptosis is a normal and orderly program of cell death, and avoiding apoptosis is an essential element for the survival of tumor cells. Several polysaccharides could inhibit HCC growth by apoptosis, but their targets on apoptosis of tumor cells were different. Caspase plays a key role in the mechanism of apoptosis and is the initiator of cell apoptosis. *Grifola frondose* polysaccharide directly increased apoptosis of HCC cells through mitochondria apoptotic pathway in a caspases-dependent pattern (Yu et al., 2020). The polysaccharide extracted from *Murus alba* L. prevented HCC progression through the induction of apoptosis, as evidenced by a reduction in the levels of B-cell lymphoma-2 (Bcl-2) and an increase in the levels of Bcl-2-associated X protein (Bax), caspase3 and caspase9 (Li et al., 2021). As another critical regulator of apoptosis, cytochrome c initiates the cell death via apoptosis activating factor (Kalpage et al., 2019). *Pleurotus ostreatus* polysaccharide exerted anti-tumor effects through inducing apoptosis by participating in the regulation of cytochrome c (Khinsar et al., 2021). Micro ribonucleic acids (RNAs) were reported to be aberrantly expressed in many cancers, and involved in cellular processes such as apoptosis, proliferation, and differentiation (Wilk and Braun, 2018). Polysaccharide from *Rhizopus nigricans* inhibited HCC proliferation and promoted HCC apoptosis through the miR-494-3p/tripartite motif containing 36 (TRIM36) axis (Yan et al., 2022). Both of undeproteinized purified polysaccharide and papain-deproteinized polysaccharide from *Sophora japonica* L. attenuated the migration of HCC cells and induced apoptosis, but the mechanism needed further research (Zhong et al., 2022). In recent years, iron metabolism is considered a key mechanism in many liver pathologies, and iron overload is directly related to the risk of developing HCC (Kouroumalis et al., 2023). *Dandelion* polysaccharide decreased iron burden in hepatoma cells and grafted tumors of mice model via JAK/STAT pathway, thereby inhibiting HCC (Ren et al., 2021). In addition to its direct anti-tumor effect on HCC, some polysaccharides can also enhance the efficacy of other anti-tumor drugs through synergistic effects. *Astragalus* polysaccharide promoted doxorubicin-induced apoptosis of HCC cells through reducing the O-GlcNAcylation (Li M. et al., 2023). Neutral *Panax notoginseng* polysaccharide significantly increased anti-tumor effects of cyclophosphamide in H22 tumor-bearing mice via the cellular and humoral immunity (Liu X. et al., 2021).

In summary, polysaccharides can exert anti-HCC effects through regulating immunity, preventing cancer cell metastasis, blocking angiogenesis, inducing apoptosis, targeting Mirco RNAs and reducing iron load. The therapeutic effects of polysaccharides on HCC have been widely recognized at cellular and animal levels. However, their efficacy still needs further evaluation in human clinical trials. The drugs commonly used in clinical HCC treatment have limited efficacy and high adverse reactions. Polysaccharides hold great promise as adjuvant drugs because they can synergistically enhance the effects of other antitumor drugs, reduce adverse reactions, and improve the quality of life of patients. However, the low bioavailability of polysaccharides needs to be solved urgently, which is associated with the high molecular weight and complex structure. Adequate structural characterization and studies on the structure-activity relationship, as well as the achievement of rational structural optimization of polysaccharides, will be effective approaches to solve this problem.

## 4 Application of polysaccharides as drug carrier in liver diseases

To date, the materials typically used in drug delivery systems are mostly artificially synthesized polymers, which are prone to poor metabolism and harmful metabolites in the body (Meng et al., 2022). Polysaccharides, as an important constituent of diet, decompose into innocuous degradation products in the body, and is commodious for structural modification (Zhang H. et al., 2022). Furthermore, a wide range of polysaccharide sources, including plants, animals, and fungi, are conducive to cost control. Polysaccharides are emerging as a drug carrier as an alternative to polymer materials. The studies of polysaccharides as drug carriers in liver diseases are shown in Table 7.

**TABLE 7:** Polysaccharides as drug carrier in liver diseases.

Carrier type	Polysaccharide	Source	Loaded drug	Modified material	Liver disease	Model	References
Nanoparticles	*Angelica sinensis* polysaccharide	*Angelica sinensis* (Oliv.) Diels	Doxorubicin	Deoxycholic acid	Liver cancer	HepG2-bearing nude mice	Zhang S. L. et al. (2019)
Micelles	*Bletilla striata* polysaccharide	*Bletilla striata* (Thunb.) Reichb. f.	Docetaxel	Stearic acid, cystamine	Liver cancer	HepG2, 4 T1 cells	Liu et al. (2020)
Nanoparticles	*Angelica sinensis* polysaccharide	*Angelica sinensis* (Oliv.) Diels	Curcumin	Cholesteryl hemisuccinate	ALD	Alcohol-induced BALB/c	Wang et al. (2020a)
Nanomicelle	*Angelica* polysaccharide	*Angelica sinensis* (Oliv.) Diels	Curcumin	Glycyrrhetic acid	Liver cancer	HepG2-bearing nude mice	Guo et al. (2021)
Micelles	*Angelica sinensis* polysaccharide	*Angelica sinensis* (Oliv.) Diels	Curcumin	Arachidonic acid	Liver cancer	HepG2	Liu Y. H. et al. (2021)
Nanoparticles	*Angelica sinensis* polysaccharide	*Angelica sinensis* (Oliv.) Diels	Oridonin	Deoxycholic acid	Liver cancer	H22-bearing BALB/c	Sun et al. (2024)

Besides surgical resection and transplantation, chemotherapy drugs play a dominant role in cancer treatment strategies. However, chemotherapy drugs have limitations due to high toxicity, non-selectivity, and side effects. Compared to conventional chemotherapy, nanocarriers could significantly improve therapeutic efficacy and reduce systemic toxicity (Dubey et al., 2022). As a potent anticancer drug, doxorubicin (Dox) has been widely applied in clinical chemotherapy of HCC, accompanied by an increased risk of toxic side effects. A Dox-carrying nanoparticle of *A. sinensis* polysaccharide modified with deoxycholic acid inhibited the growth of human hepatocellular carcinoma G2 (HepG2) tumor to a greater extent than free Dox and reduced the toxicity of Dox, especially cardiac toxicity (Zhang S. L. et al., 2019). *A Bletilla striata* polysaccharide-based copolymer self-assembled into micelles and encapsulated docetaxel, which exhibited a redox- and pH-dual responsive characteristics and enhanced the inhibition of HCC cells (Liu et al., 2020).

In addition to chemotherapy drugs, polysaccharides can be used as carriers to deliver small molecule drugs. Curcumin (Cur) with low solubility in aqueous media, fast degradation and poor oral bioavailability is a hepatoprotective polyphenolic compound. The nano delivery system based on polysaccharides can overcome the main drawbacks of Cur. Compared with free Cur, the Cur-loaded *A. sinensis* polysaccharide nanoparticles showed higher solubility, good photostability, and better anti-ALD effect (Wang et al., 2020a). There are numerous asialoglycoprotein receptor in the hepatocyte surface, which shows high specific appetency for glucose, N-acetylgalactosamine and galactose, and it is a specific target for hepatocytes. Zhang et al. found that *A. sinensis* polysaccharide containing a lot of galactose and glucose had a high affinity for asialoglycoprotein receptor (Zhang et al., 2018). *Angelica sinensis* polysaccharide-based nanocarriers modified by glycyrrhetic acid and arachidonic acid have been developed for the targeted liver delivery of Cur (Guo et al., 2021; Liu Y. H. et al., 2021). These nanocarriers could increase drug concentration at the tumor site and provide synergistic effects against HCC. Moreover, an oridonin-loaded *A. sinensis* polysaccharide nanoparticles with pH-dependent drug release characteristic could increase HepG2 cell apoptosis and inhibit tumor growth in hepatoma 22 (H22) tumor-bearing mice (Sun et al., 2024).

**FIGURE 2:**
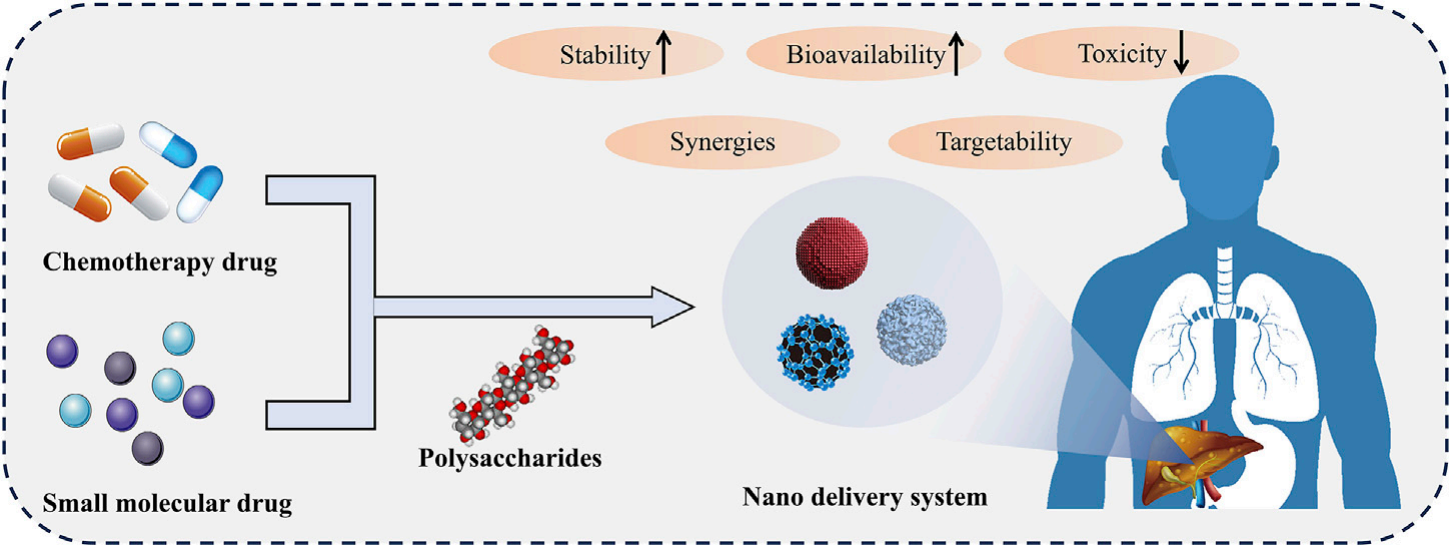
Polysaccharides based drug delivery system. The nano delivery system composed of polysaccharides delivers chemotherapy drugs or small molecule drugs to the liver, which can improve drug stability, bioavailability, and reduce toxicity. In addition, polysaccharides work synergistically with drugs to enhance their efficacy.

Overall, polysaccharides have great potential as drug carriers for chemotherapy drugs and small molecule drugs in liver diseases (Figure 2). The utilization of polysaccharides targeting liver characteristics could make it possible to deliver drugs to the target site. Polysaccharides-based drug delivery systems could operate drug release under specific conditions or sustained-release, facilitating more precise control of drug concentration and reducing toxic side effects. In addition, polysaccharides with hepatoprotective effects could synergize with loaded hepatoprotective drugs to achieve better therapeutic effects.

## 5 Discussion

Liver diseases are a worldwide public health challenge with persistently high mortality (Devarbhavi et al., 2023). However, the existing hepatoprotective drugs have limited efficacy and side effects. Polysaccharides have various biological activities such as antioxidant, anti-inflammatory, and immune regulation (Yu C. et al., 2023; Lai et al., 2024; Lu et al., 2025). Recently, nontoxic polysaccharides from plants, fungi, and other sources have been reported to exert protective effects against DILI, ALD, hepatitis B, NAFLD, liver fibrosis, and HCC. Therefore, polysaccharides are considered as a promising natural biomacromolecule for treating liver diseases.

**FIGURE 3:**
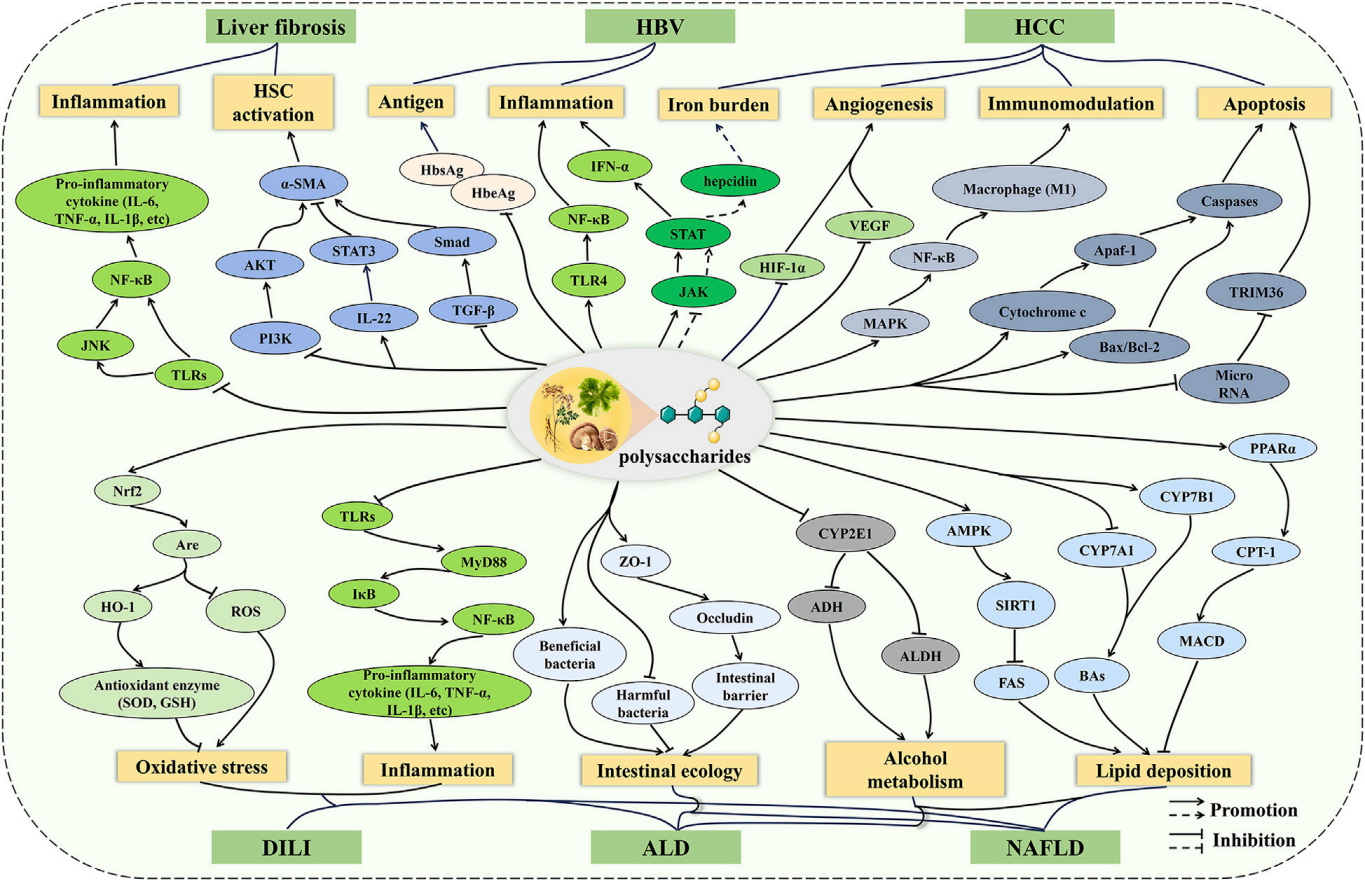
The mechanism of polysaccharides on liver diseases. The preventive and therapeutic effects of polysaccharides on liver diseases involve multiple pathways, including inflammation, oxidative stress, metabolic regulation, intestinal ecology, apoptosis, and so on. Polysaccharides do not play a role through a single channel, but the result of various mechanisms.

The underlying mechanisms of liver diseases are complicated, involving oxidative stress, inflammation, immune imbalance, and apoptosis (Neshat et al., 2021). It is gratifying that the pharmacological effects of polysaccharides are multifaceted, meaning that polysaccharides do not play a role through a single channel, but the result of various mechanisms (Du et al., 2023; Duan et al., 2024). Therefore, polysaccharides exhibit a range of bioactivities through multi-target and multi-pathway strategies, which are of great significance for the treatment of liver diseases involving complex mechanisms. In this review, recent studies on the effects of polysaccharides on liver diseases are summarized, and the potential mechanisms are described (Figure 3). Overall, the protective mechanisms of polysaccharides against liver diseases can be roughly divided into five points. (1) Inflammation and oxidative stress play important roles in the development of liver diseases, and polysaccharides regulate inflammatory cytokines through pathways such as TLRs/NF-κB and JAK/STAT to balance inflammatory responses in DILI, ALD, HBV, NAFLD, and liver fibrosis. Polysaccharides alleviate oxidative stress through the Nrf2/ARE pathway in DILI, ALD, and NAFLD. (2) In ALD and NAFLD, polysaccharides improve alcohol metabolism and lipid deposition by regulating the CYPs and lipid metabolism pathways. In addition, polysaccharides maintain metabolic balance through the regulation of gut microbiota and the repair of intestinal barrier function. (3) Polysaccharides inhibit the activation of HSC via the TGF-β/Smad, IL-22/STAT3, and PI3K/AKT pathways, thus improving ECM accumulation during liver fibrosis. (4) Polysaccharides inhibit tumor growth by promoting apoptosis, alleviating the iron burden, and suppressing angiogenesis, as well as exerting anti-tumor effects by regulating macrophage polarization through the MAPK pathway. (5) Polysaccharides inhibit HBV replication and activity by inhibiting antigen secretion. However, despite the potential hepatoprotective effects and mechanisms of polysaccharides have been demonstrated in numerous cellular and animal models, their efficacy and safety still require further evaluation in clinical studies of liver diseases. In addition, many polysaccharides exhibit low bioavailability due to their large molecular weight and poor solubility, which hinders the development of innovative drugs and formulations based on polysaccharides (Zheng et al., 2020). To overcome this limitation, physical modification and nanomedicine may serve as promising approaches to enhance bioavailability.

In addition to their pharmacological role, polysaccharides have been applied in delivery systems for chemotherapy drugs and small molecule drugs, which can improve drug stability and bioavailability while reducing toxicity (Yadav et al., 2022). This indicates their considerable potential in the treatment of liver diseases. However, its application to the human body requires further evaluation. Another drawback of polysaccharides in drug delivery is the difficulty in determining the structure of polysaccharides derived from natural sources. The polysaccharide structure of the same plant has varied in different studies. Therefore, the quality standardization of polysaccharides is important for the development of polysaccharides and dosage types. This is essential to ensure the repeatability of the polysaccharide findings. The implementation of quality standardization largely depends on the uniformity of polysaccharide extraction processes and raw material supplies.

In general, the bioactivity of polysaccharides is closely related to their structure. The complexity of polysaccharides makes it difficult to analyze their structure in detail. At present, structural studies of polysaccharides mainly focused on molecular weight, monosaccharide composition, and simple primary structures, with a few identified repetitive units. A few studies have commenced exploring the structure-activity relationships of polysaccharides. Research has shown that the content of uronic acid in polysaccharides may be positively correlated with their biological activity (Qu J. et al., 2020). The polysaccharide of *P. multiflorum* Thunb in the raw with more uronic acid exhibited better improvement in liver injury than the polysaccharide of *P. multiflorum* Thunb in processed products (Wang Z. et al., 2023). There is a relationship between the composition and ratio of monosaccharides and the activity of polysaccharides. It is generally believed that polysaccharides with more complex monosaccharide compositions exhibit better hepatoprotective activities (Qu H. et al., 2020; Wang et al., 2024a). In addition, the influence of molecular weight on the hepatoprotective effects of polysaccharides is not simply a positive or negative correlation (Wang Z. et al., 2023). Two polysaccharides with different molecular weights were isolated from *Sophora tonkinensis* Gagnep., and the polysaccharide with lower molecular weight presented stronger hepatoprotective effects than the other (Cai et al., 2018). However, among the three polysaccharides isolated from *Anoectochilus roxburghii* (Wall.) Lindl, the polysaccharide with the highest molecular weight exhibited the strongest hepatoprotective effect (Zeng et al., 2016). Therefore, the relationship between molecular weight and the hepatoprotective activity of polysaccharides is complex and requires further investigation. To date, the structure-activity relationships of polysaccharides have not been completely elucidated. Further researches are required to achieve rational structural optimization of polysaccharides in order to enhance bioavailability and efficacy. This is essential for understanding the specific molecular targets and mechanisms of polysaccharides, and provides a foundation for further clinical trials and the development of innovative drugs.

Chemical modification is a method for improving the activity of polysaccharides, and the common methods include sulfonation, acetylation, phosphorylation, and selenization (Li et al., 2024; Wang et al., 2024b). It has been demonstrated that the hepatoprotective effects of polysaccharides increase after structural modification. For example, compared to the natural polysaccharide, selenized *Medicago sativa* L. polysaccharide significantly enhanced the antioxidant and anti-HCC effects (Gao et al., 2020). In addition, biological modification is a good strategy for polysaccharide structural modification (Wang et al., 2024c). Fermenting the *Lilium davidii* var. Unicolor Cotton polysaccharide with *Lactobacillus plantarum* can enhance its antioxidant activity (Song X. et al., 2021). Therefore, various modification methods can be applied in future studies to improve the hepatoprotective effects of polysaccharides. Meanwhile, the potential adverse effects of structural modifications on the physical properties and bioactivity of polysaccharides should also be noted. It is beneficial for the development of polysaccharides to identify safe and efficient structural modification strategies.

## 6 Conclusion

Polysaccharides demonstrate extensive potential in the prevention and treatment of liver diseases. The present work summarizes the hepaprotective effects, potential mechanisms, and drug carrier applications of polysaccharides based on literature in the last 6 years. The results indicate the effectiveness of polysaccharides in various liver diseases, including DILI, ALD, hepatitis B, NAFLD, liver fibrosis, and HCC. Polysaccharides exert hepatoprotective effects through the combined actions of multiple mechanisms. In addition, nanomaterials based on polysaccharides can be used for the targeted delivery of chemotherapeutic drugs and small-molecule drugs, exerting synergistic therapeutic effects. Therefore, polysaccharides demonstrate characteristics of multi-target, multi-pathway, and synergistic effects in the treatment of liver diseases, granting them unique advantages. However, further clinical trials are required to apply polysaccharides as drugs or nanocarriers in humans. Additionally, the relationship between the bioactivity and structure of polysaccharides needs to be further clarified, which will contribute to the development of drugs and formulations based on polysaccharides for liver diseases.

## 7 Perspectives

Polysaccharides, with a wide range of sources, significant biological activities, and low toxicity, have attracted more and more attention in recent years, especially the effects of liver protection. However, several problems should be considered in future studies of polysaccharides in liver diseases. First, improving the bioavailability of polysaccharides and exploring their interactions with cell surface receptors are crucial, as this will provide a theoretical basis for drug development and targeted drug design based on polysaccharides. Second, further exploration of their structure-activity relationships is needed to achieve rational structural optimization. Finally, establishing a comprehensive quality standard for polysaccharides and their derived drugs for clinical trials is essential to promote clinical trials and evaluate the safety and efficacy of polysaccharides in liver diseases. With advancements in science and technology, we believe these challenges will be addressed. Continued exploration of polysaccharides holds great potential for developing treatment strategies for liver diseases.
