# Supplementary material for: Recent advances in the role of polysaccharides in liver diseases: a review
Source: Front Pharmacol. 2025 Mar 27;16:1535717. doi: 10.3389/fphar.2025.1535717 (PMC11982827; doi:10.3389/fphar.2025.1535717)
Supplement: Supplementary file 1 [file DataSheet1.pdf]

Supplementary Table1 Summary of all retrieved pharmacological documents

Type	Polysaccharide	Sources	Type of extract	Model	Dosage	Control	References
Plant	SCLP	*Smilax china* L.	Tuberous root	APAP-induced mice	300 and 600 mg/kg (i.g.)	Saline	(Wang et al., 2022)
	SCAP	*Schisandra chinensis* (Turcz.) baill	Fruit	APAP-induced mice	10, 20, 40 mg/kg (i.g.)	Saline	(Che et al., 2019)
	PPMPs	*Polygonum multiflorum* Thunb	Tuberous root	APAP-induced mice	100 and 200 mg/kg (i.g.)	Saline	(Wang et al., 2023)
	RPMPs	*Polygonum multiflorum* Thunb	Tuberous root	APAP-induced mice	100 and 200 mg/kg (i.g.)	Saline	(Wang et al., 2023)
	PVSP	*Prunella vulgaris* L.	Aerial part	INH-induced mice	100 mg/kg (i.g.)	Saline	(Wang et al., 2021)
	EPPS	*Echinacea purpurea* (L.) Moench	Tuberous root	APAP-induced mice	20, 50 mg/kg (i.g.)	Saline	(Yu et al., 2022)
	EP	*Enteromorpha prolifera*	Whole plant	Alcohol-induced mice	50, 100 mg/kg (i.g.)	Saline, Silymarin	(Yan et al., 2024)
	ASP	*Angelica sinensis (Oliv.)* Diels	Tuberous root	Alcohol-induced mice	100, 300 mg/kg (i.g.)	Saline	(He et al., 2022)
				Alcohol-induced AML12 cells	100, 300 μg/ml	Distilled water	
	PFPs	*Polygala fallax* Hemsl	Tuberous root	HFD and alcohol-induced mice	50, 100, 200 mg/kg (i.g.)	Saline, Silymarin	(Lv et al., 2024)
				Alcohol-induced HepG2 cells	100 μg/ml	Distilled water	
	PNP80b-2	*Pinus koraiensis* Sieb. et Zucc.	Fruit	Alcohol-induced mice	200, 400 mg/kg (i.g.)	Saline, Bifendate	(Qu et al., 2020)
	DOP	*Dendrobium officinale* Kimura et Migo	Aerial part	Alcohol-induced Rats	100, 400 mg/kg (i.g.)	Saline, Silymarin	(Yang et al., 2020)
	EPP80	*Echinacea purpurea* (L.) Moench	Aerial part	Alcohol-induced mice	100, 200, 400 mg/kg (i.g.)	Saline, Bifendate	(Jiang et al., 2021)
	AHPN80	*Alhagi sparsifolia* Shap	Aerial part	Alcohol-induced mice	200, 600 mg/kg (i.g.)	Saline, Silymarin	(Song et al., 2024)
				H_2O_2-induced HepG2 cells	50, 100, 200 μg/ml	Distilled water	
	SCP	*Schisandra chinensis*	Fruit	Alcohol-induced mice	14.78, 29.55, 59.1 mg/kg (i.g.)	Saline	(Chi et al., 2024)

		(Turcz.) baill					
	NCVP	*Nostoc commune* Vauch.	Whole plant	Alcohol-induced mice	100, 200, 400 mg/kg (i.g.)	Saline, Silymarin	(Yang et al., 2023)
	RIP	*Isatis indigotica* Fort.	Tuberous root	HepG2.2.15 cells	50, 100, 200 μg/ml	Culture medium, Lamivudine	(Wang et al., 2020)
	VCP	*Viscum coloratum* （K om.） Nakai	Aerial part	HepG2.2.15 cells	1, 10, 100, 1000 μg/ml	Culture medium, Lamivudine	(Chai et al., 2019)
	FP-1	*Linum usitatissimum* L.	Aerial part	HepG2.2.15 cells	62.5, 125, 250, 500, 1000 μg/ml	Culture medium, Lamivudine	(Liang et al., 2019)
	SLP-4	*Saussurea laniceps* Hand. -Mazz.	Aerial part	HepG2.2.15 cells	62.5, 125, 250, 500, 1000 μg/ml	Culture medium, Lamivudine	(Chen et al., 2019)
	ASP	*Angelica sinensis (Oliv.)* Diels	Tuberous root	HFD-induced mice	160 mg/kg (i.g.)	Saline	(Luo et al., 2023)
	APS	*Astragalus membranaceus* (Fisch.) bunge.	Tuberous root	HFD-induced mice	8% (p.o.)	Normal HFD diet	(Zheng et al., 2024)
	CLP	*Chaetomorpha linum*	Whole plant	HFD-induced mice	50, 150 mg/kg (i.g.)	Saline	(Chu et al., 2022)
	LBP	*Lycium barbarum* L.	Fruit	HFD-induced Rats	50 mg/kg (p.o.)	Saline	(Gao et al., 2021)
	MDG	*Ophiopogon japonicus*	Tuberous root	HFD-induced mice	5%, 8% (p.o.)	Normal HFD diet	(Zhang et al., 2022)
	SMRR	*Salviae miltiorrhizae* Bunge	Fibrous root	HFD-induced mice	10, 20 mg/kg (i.g.)	Saline	(Li et al., 2022)
	PCP1	*Polygonatum cyrtonema* Hua	Tuberous root	HFD-induced mice	200, 400 mg/kg (i.g.)	Saline	(Liu et al., 2022)
	CPP	*Crataegus pinnatifida* Bunge	Fruit	HFD-induced mice	100, 250, 500 mg/kg (i.g.)	Saline	(Hao et al., 2024)
	PAMK	*Atractylodes macrocephala* Koidz.	Tuberous root	HFD-induced mice	200 mg/kg (p.o.)	Normal HFD diet	(Chen et al., 2024)
	YJ3A1	*Rosa chinensis* Jacq.	Aerial part	CCl_4-induced mice	25, 50 mg/kg (i.g.)	Saline, Obeticholic acid	(Jing et al., 2024)
	CPP-1	*Codonopsis pilosula*	Fibrous root	HFD-induced mice	180 mg/kg (i.g.)	Saline, Simvastatin	(Ma et al., 2024)

		(Franch.) Nannf.					
	SP	*Hippophae rhamnoides* L.	Fruit	HFD-induced Rats	200 mg/kg (i.g.)	Saline, berberine hydrochloride	(Yan et al., 2024)
	APS	*Astragalus membranaceus* (Fisch.) Bge.	Tuberous root	Alcohol-induced Rats	200, 400 mg/kg (i.g.)	Saline	(Sun et al., 2023)
	BPS	*Bletilla striata* (Thunb.) Reichb. f	Tuberous root	CCl_4-induced mice	75, 150, 300 mg/kg (i.g.)	Saline, Silymarin	(Jiang et al., 2023)
	DOP	*Dendrobium officinale* Kimura et Migo	Tuberous root	CCl_4-induced Rats	200, 400, 800 mg/kg (i.g.)	Saline	(Wang et al., 2020)
	CPP-A-1	*Codonopsis pilosula* (Franch) Nannf.	Fibrous root	CCl_4-induced mice	50, 100 mg/kg (i.g.)	Saline, Colchicine	(Meng et al., 2023)
	AMP	*Aronia melanocarpa* (Michx.) Elliott	Fruit	TAA-induced mice	200, 400 mg/kg (i.g.)	Saline, Colchicine	(Zhao et al., 2022)
	ASP	*Angelica sinensis* (Oliv.) Diels	Tuberous root	CCl_4-induced mice	200 mg/kg (i.g.)	Saline	(Wang et al., 2020)
	ABWW	*Achyranthes bidentata* Bl	Tuberous root	CCl_4-induced mice	100, 200 mg/kg (i.p.)	Saline, Obeticholic acid	(Dai et al., 2024)
	BPS	*Ocimum basilicum* L.	Aerial part	MHCC97H tumor bearing mice	100, 200, 400 mg/kg (i.g.)	Saline, Sorafinib	(Feng et al., 2019)
	ASP	*Asparagus officinalis* L.	Whole plant	SK-Hep1and Hep-3B cells	1.25, 2.5, 5, 10, 20, 30 mg/ml	Culture medium	(Cheng et al., 2019)
	MEP	*Morus alba* L.	Fruit	DEN/PB-induced Rats	50, 100 mg/kg (i.g.)	Saline	(Li et al., 2021)
	PFS-1	*Sophora japonica* L.	Fruit	SMMC 7721 cells	125, 250, 500, 1000, 2000 μg/ml	Culture medium, Docetaxel	(Zhong et al., 2022)
	PFS-2	*Sophora japonica* L.	Fruit	SMMC 7721 cells	125, 250, 500, 1000, 2000 μg/ml	Culture medium, Docetaxel	(Zhong et al., 2022)
	DP	*Taraxaci Herba*	Fibrous root	Hepa1-6 or H22 tumor bearing mice	200 mg/kg (i.p.)	Saline	(Ren et al., 2021)
	APS	*Astragalus*	Tuberous root	Hep3B tumor bearing	50 mg/kg (i.p.)	Saline,	(Li et al.,

		membranaceus (Fisch.) Bge.		mice		Doxorubicin	2023)
	NPPN	*Panax notoginseng* (Burk.) F.H.Chen	Fibrous root	H22 tumor bearing mice	93, 188, 375 mg/kg (i.g.)	Saline, Cyclophosphamide	(Liu et al., 2021)
Fungi	Phps	*Phellinus linteus*	Mycelia	APAP-induced mice	40, 60, 80 mg/kg (i.p.)	Saline	(Zhao et al., 2022)
	PL-N1	*Phellinus linteus*	Mycelia	APAP-induced mice	10, 50, 100 mg/kg (i.g.)	Saline, Silibinin	(Chen et al., 2020)
	PFP-1	*Pleurotus geesteranus*	Mycelia	Alcohol-induced mice	400 mg/kg (i.g.)	Saline, Bifendate	(Song et al., 2021)
	MEP	*Morchella esculenta*	Mycelia	Alcohol-induced mice	50, 100 mg/kg (i.g.)	Saline, Silymarin	(Teng et al., 2023)
	PSP-1b1	*Coriolus versicolor*	Mycelia	Alcohol-induced mice	80, 160 mg/kg (i.g.)	Saline, Silymarin	(Wang et al., 2019)
	PCP-1C	*Poria cocos* (Schw.) Wolf	Sclerotium	Alcohol-induced mice	25, 50, 100 mg/kg (i.g.)	Saline, Bifendate	(Jiang et al., 2022)
	ORPS	*Oudemansiella raphanipies*	Mycelia	HFD-induced mice	50, 100, 200 mg/kg (i.g.)	Saline	(Jiang et al., 2022)
	CVP	*Coriolus versicolor*	Mycelia	HFD-induced mice	100, 400 mg/kg (i.g.)	Saline, Atorvastatin	(Tang et al., 2023)
	PCP	*Poria cocos* (Schw.) Wolf	Sclerotium	HFD-induced mice	50, 100, 200 mg/kg (i.g.)	Saline	(Ye et al., 2022)
	SSP	*Sagittaria sagittifolia* L.	Mycelia	MCD-induced mice	0.8 g/kg (p.o.)	Saline	(Deng et al., 2020)
	ASRP	*Stropharia rugoso-annulata*	Mycelia	HFD-induced mice	400 mg/kg (i.g.)	Saline, Simvastatin	(Li et al., 2022)
	GLP	*Ganoderma lucidum* (Curtis) P. Karst.	Mycelia	CCl_4-induced mice	150, 300 mg/kg (i.g.)	Saline, Colchicine	(Chen et al., 2023)
	CP	*Coprinus comatus* (Muell.:Fr.) Gray	Mycelia	CCl_4-induced mice	200, 400 mg/kg (i.g.)	Saline	(Zhao et al., 2022)
				LPS-induced LX-2 cells	60, 120, 240 μg/ml	Phosphate buffered saline	
	GLPS	*Ganoderma lucidum* (Curtis) P. Karst.	Mycelia	Hepa1-6 tumor bearing mice	50, 100, 200 mg/kg (i.g.)	Saline	(Li et al., 2023)
				Hepa1-6 cells	200 μg/ml	Culture medium	
	GFAP	*Grifola frondosa* (Dicks.) Gray	Mycelia	H22 and HepG2 cells	12.5, 25, 50, 100, 200, 400, 600, 800 μg/ml	Culture medium	(Yu et al., 2020)

	POP	*Pleurotus ostreatus*	Mycelia	H22 tumor bearing mice	75, 150, 300 mg/kg (i.p.)	Saline, Cyclophosphamide	(Khinsar et al., 2021)
				HepG2 and HCCLM3 cells	300, 400, 500 μg/ml	Culture medium	
	EPS1-1	*Rhizopus nigrum*	Mycelia	HepG2 and HuH7 cells	25, 50, 100, 200 μg/ml	Culture medium	(Yan et al., 2022)
Animal	SEP	*Strongylocentrotus nudus*	Egg	HBV-transgene mice	40 mg/kg (i.p.)	Saline, Lamivudine	(Yu et al., 2023)
				HepG2.2.15 cells	100, 200, 400, 800 μg/ml	Culture medium, Lamivudine	

Supplementary Table 2 Abbreviations

ADH	alcohol dehydrogenase
AKT	protein kinase B
ALD	alcoholic liver disease
ALT	alanine aminotransferase
AMPK	AMP-activated protein kinase
APAP	acetaminophen
ARE	antioxidant response element
AST	aspartate aminotransferase
Bax	BCL-2-associated X
Bcl-2	B-cell lymphoma-2
CAT	catalase
CPT1	carnitine palmitoyltransferase-1
Cur	curcumin
CYP	cytochrome P450 proteins
DNA	deoxyribonucleic acid
DILI	drug-induced liver injury
Dox	doxorubicin
ECM	extracellular matrix
FAK	focal adhesion kinase
H22	hepatoma 22
HBV	hepatitis B virus
HBsAg	hepatitis B surface antigen
HBeAg	hepatitis Be antigen
HepG2	human hepatocellular carcinoma G2
HCC	hepatocellular carcinoma
HIF-1α	hypoxia inducible factor-1α
HO-1	heme oxygenase 1
HSC	hepatic stellate cell
IL-1β	interleukin-1β

IL-6	interleukin-6
IL-22	interleukin-22
JAK	janus kinase
JNK	c-jun N-terminal kinase
LPS	lipopolysaccharide
MAPK	mitogen-activated protein kinase
MCAD	medium-chain acyl-CoA dehydrogenase
MDA	malonaldehyde
NAFLD	Non-alcoholic fatty liver disease
NF-κB	nuclear factor-kappa B
Nrf2	nuclear factor erythroid 2-associated factor 2
PI3K	phosphoinositide 3 kinase
PTRF	polymerase I and transcript release factor
PPARα	peroxisome proliferator-activated receptor α
RNA	ribonucleic acid
ROS	reactive oxygen species
SIRT1	sirtuin 1
Smad	drosophila mothers against decapentaplegic
SOD	superoxide dismutase
STAT	signal transducer and activator of transcription
TGF-β	transforming growth factor β
TLR4	toll-like receptor 4
TNF-α	tumor necrosis factor α
TRIM36	tripartite motif containing 36
VEGF	vascular endothelial growth factor

References

Chai, Y., Kan, L., and Zhao, M. (2019). Enzymatic extraction optimization, anti-HBV and antioxidant activities of polysaccharides from Viscum coloratum (Kom.) Nakai. *Int. J. Biol. Macromol.* 134**,** 588-594. doi: 10.1016/j.ijbiomac.2019.04.173.

Che, J., Yang, S., Qiao, Z., Li, H., Sun, J., Zhuang, W., et al. (2019). Schisandra chinensis acidic polysaccharide partialy reverses acetaminophen-induced liver injury in mice. *J. Pharmacol. Sci.* 140(3)**,** 248-254. doi: 10.1016/j.jphs.2019.07.008.

Chen, C., Chen, J., Wang, Y., Fang, L., Guo, C., Sang, T., et al. (2023). Ganoderma lucidum polysaccharide inhibits HSC activation and liver fibrosis via targeting inflammation, apoptosis, cell cycle, and ECM-receptor interaction mediated by TGF-β/Smad signaling. *Phytomedicine* 110**,** 154626. doi: 10.1016/j.phymed.2022.154626.

Chen, C., Liu, X., Qi, S., A, C.P.D., Yan, J., and Zhang, X. (2020). Hepatoprotective effect of Phellinus linteus mycelia polysaccharide (PL-N1) against acetaminophen-induced liver injury in mouse. *Int. J. Biol. Macromol.* 154**,** 1276-1284. doi: 10.1016/j.ijbiomac.2019.11.002.

Chen, J., Yang, S., Luo, H., Fu, X., Li, W., Li, B., et al. (2024). Polysaccharide of Atractylodes macrocephala Koidz alleviates NAFLD-induced hepatic inflammation in mice by modulating the TLR4/MyD88/NF-kappaB pathway. *Int. Immunopharmacol.* 141**,** 113014. doi: 10.1016/j.intimp.2024.113014.

Chen, W., Zhu, X., Ma, J., Zhang, M., and Wu, H. (2019). Structural Elucidation of a Novel Pectin-Polysaccharide from the Petal of Saussurea laniceps and the Mechanism of its Anti-HBV Activity. *Carbohydr. Polym.* 223**,** 115077. doi: 10.1016/j.carbpol.2019.115077.

Cheng, W., Cheng, Z., Xing, D., and Zhang, M. (2019). Asparagus Polysaccharide Suppresses the Migration, Invasion, and Angiogenesis of Hepatocellular Carcinoma Cells Partly by Targeting the HIF-1alpha/VEGF Signalling Pathway In Vitro. *Evid Based Complement. Alternat. Med.* 2019**,** 3769879. doi: 10.1155/2019/3769879.

Chi, Y.Y., Xiang, J.Y., Li, H.M., Shi, H.Y., Ning, K., Shi, C., et al. (2024). Schisandra chinensis polysaccharide prevents alcohol-associated liver disease in mice by modulating the gut microbiota-tryptophan metabolism-AHR pathway axis. *Int. J. Biol. Macromol.* 282(Pt 2)**,** 136843. doi: 10.1016/j.ijbiomac.2024.136843.

Chu, X., Zhou, Y., Zhang, S., Liu, S., Li, G., and Xin, Y. (2022). Chaetomorpha linum polysaccharides alleviate NAFLD in mice by enhancing the PPARalpha/CPT-1/MCAD signaling. *Lipids Health Dis.* 21(1)**,** 140. doi: 10.1186/s12944-022-01730-x.

Dai, X., Du, Z., Jin, C., Tang, B., Chen, X., Jing, X., et al. (2024). Inulin-like polysaccharide ABWW may impede CCl(4) induced hepatic stellate cell activation through mediating the FAK/PI3K/AKT signaling pathway in vitro & in vivo. *Carbohydr. Polym.* 326**,** 121637. doi: 10.1016/j.carbpol.2023.121637.

Deng, X., Ke, X., Tang, Y., Luo, W., Dong, R., Ge, D., et al. (2020). Sagittaria sagittifolia polysaccharide interferes with arachidonic acid metabolism in non-alcoholic fatty liver disease mice via Nrf2/HO-1 signaling pathway. *Biomed. Pharmacother.* 132**,** 110806. doi: 10.1016/j.biopha.2020.110806.

Feng, B., Zhu, Y., Sun, C., Su, Z., Tang, L., Li, C., et al. (2019). Basil polysaccharide inhibits hypoxia-induced hepatocellular carcinoma metastasis and progression through suppression of HIF-1alpha-mediated epithelial-mesenchymal transition. *Int. J. Biol. Macromol.* 137**,** 32-44. doi: 10.1016/j.ijbiomac.2019.06.189.

Gao, L.L., Ma, J.M., Fan, Y.N., Zhang, Y.N., Ge, R., Tao, X.J., et al. (2021). Lycium barbarum polysaccharide combined with aerobic exercise ameliorated nonalcoholic fatty liver disease through restoring gut microbiota, intestinal barrier and inhibiting hepatic inflammation. *Int. J. Biol. Macromol.* 183**,** 1379-1392. doi: 10.1016/j.ijbiomac.2021.05.066.

Hao, P., Yang, X., Yin, W., Wang, X., Ling, Y., Zhu, M., et al. (2024). A study on the treatment effects of Crataegus

pinnatifida polysaccharide on non-alcoholic fatty liver in mice by modulating gut microbiota. *Front. Vet. Sci.* 11, 1383801. doi: 10.3389/fvets.2024.1383801.

He, Z., Guo, T., Cui, Z., Xu, J., Wu, Z., Yang, X., et al. (2022). New understanding of Angelica sinensis polysaccharide improving fatty liver: The dual inhibition of lipid synthesis and CD36-mediated lipid uptake and the regulation of alcohol metabolism. *Int. J. Biol. Macromol.* 207, 813-825. doi: 10.1016/j.ijbiomac.2022.03.148.

Jiang, G., Wang, B., Wang, Y., Kong, H., Wang, Y., Gao, P., et al. (2023). Structural characteristics of a novel Bletilla striata polysaccharide and its activities for the alleviation of liver fibrosis. *Carbohydr. Polym.* 313, 120781. doi: 10.1016/j.carbpol.2023.120781.

Jiang, H., Zhu, H., Huo, G., Li, S., Wu, Y., Zhou, F., et al. (2022). Oudemansiella raphanipies Polysaccharides Improve Lipid Metabolism Disorders in Murine High-Fat Diet-Induced Non-Alcoholic Fatty Liver Disease. *Nutrients* 14(19), 4092. doi: 10.3390/nu14194092.

Jiang, W., Zhu, H., Xu, W., Liu, C., Hu, B., Guo, Y., et al. (2021). Echinacea purpurea polysaccharide prepared by fractional precipitation prevents alcoholic liver injury in mice by protecting the intestinal barrier and regulating liver-related pathways. *Int. J. Biol. Macromol.* 187, 143-156. doi: 10.1016/j.ijbiomac.2021.07.095.

Jiang, Y.H., Wang, L., Chen, W.D., Duan, Y.T., Sun, M.J., Huang, J.J., et al. (2022). Poria cocos polysaccharide prevents alcohol-induced hepatic injury and inflammation by repressing oxidative stress and gut leakiness. *Front. Nutr.* 9, 963598. doi: 10.3389/fnut.2022.963598.

Jing, X., Zhou, G., Zhu, A., Jin, C., Li, M., and Ding, K. (2024). RG-I pectin-like polysaccharide from Rosa chinensis inhibits inflammation and fibrosis associated to HMGB1/TLR4/NF-kappaB signaling pathway to improve non-alcoholic steatohepatitis. *Carbohydr. Polym.* 337, 122139. doi: 10.1016/j.carbpol.2024.122139.

Khinsar, K.H., Abdul, S., Hussain, A., Ud Din, R., Lei, L., Cao, J., et al. (2021). Anti-tumor effect of polysaccharide from Pleurotus ostreatus on H22 mouse Hepatoma ascites in-vivo and hepatocellular carcinoma in-vitro model. *AMB Express* 11(1), 160. doi: 10.1186/s13568-021-01314-5.

Li, G.L., Tang, J.F., Tan, W.L., Zhang, T., Zeng, D., Zhao, S., et al. (2023). The anti-hepatocellular carcinoma effects of polysaccharides from Ganoderma lucidum by regulating macrophage polarization via the MAPK/NF-kappaB signaling pathway. *Food Funct.* 14(7), 3155-3168. doi: 10.1039/d2fo02191a.

Li, L., Lan, X., Peng, X., Shi, S., Zhao, Y., Liu, W., et al. (2022). Polysaccharide from Salviae miltiorrhizae Radix et Rhizoma Attenuates the Progress of Obesity-Induced Non-Alcoholic Fatty Liver Disease through Modulating Intestinal Microbiota-Related Gut-Liver Axis. *Int. J. Mol. Sci.* 23(18), 10620. doi: 10.3390/ijms231810620.

Li, M., Duan, F., Pan, Z., Liu, X., Lu, W., Liang, C., et al. (2023). Astragalus Polysaccharide Promotes Doxorubicin-Induced Apoptosis by Reducing O-GlcNAcylation in Hepatocellular Carcinoma. *Cells* 12(6), 866. doi: 10.3390/cells12060866.

Li, S., Li, Y., Sun, H., Jiang, Y., Pan, K., Su, Y., et al. (2021). Mulberry fruit polysaccharides alleviate diethylnitrosamine/phenobarbital-induced hepatocarcinogenesis in vivo: the roles of cell apoptosis and inflammation. *Bioengineered* 12(2), 11599-11611. doi: 10.1080/21655979.2021.1993716.

Li, X., Cui, W., Cui, Y., Song, X., Jia, L., and Zhang, J. (2022). Stropharia rugoso-annulata acetylated polysaccharides alleviate NAFLD via Nrf2/JNK1/AMPK signaling pathways. *Int. J. Biol. Macromol.* 215, 560-570. doi: 10.1016/j.ijbiomac.2022.06.156.

Liang, S., Li, X., Ma, X., Li, A., Wang, Y., Reaney, M.J.T., et al. (2019). A flaxseed heteropolysaccharide stimulates immune responses and inhibits hepatitis B virus. *Int. J. Biol. Macromol.* 136, 230-240. doi: 10.1016/j.ijbiomac.2019.06.076.

Liu, W., Shao, T., Tian, L., Ren, Z., Gao, L., Tang, Z., et al. (2022). Structural elucidation and anti-nonalcoholic fatty

liver disease activity of Polygonatum cyrtonema Hua polysaccharide. *Food Funct.* 13(24)**,** 12883-12895. doi: 10.1039/d2fo03384d.

Liu, Y.H., Qin, H.Y., Zhong, Y.Y., Li, S., Wang, H.J., Wang, H., et al. (2021). Neutral polysaccharide from Panax notoginseng enhanced cyclophosphamide antitumor efficacy in hepatoma H22-bearing mice. *BMC Cancer* 21(1)**,** 37. doi: 10.1186/s12885-020-07742-z.

Luo, L., Zhang, H., Chen, W., Zheng, Z., He, Z., Wang, H., et al. (2023). Angelica sinensis polysaccharide ameliorates nonalcoholic fatty liver disease via restoring estrogen-related receptor alpha expression in liver. *Phytother. Res.* 37(11)**,** 5407-5417. doi: 10.1002/ptr.7982.

Lv, R., Cao, H., Zhong, M., Wu, J., Lin, S., Li, B., et al. (2024). Polygala fallax Hemsl polysaccharides alleviated alcoholic fatty liver disease by modifying lipid metabolism via AMPK. *Int. J. Biol. Macromol.* 279**,** 135565. doi: 10.1016/j.ijbiomac.2024.135565.

Ma, K., Yi, X., Yang, S.T., Zhu, H., Liu, T.Y., Jia, S.S., et al. (2024). Isolation, purification, and structural characterization of polysaccharides from Codonopsis pilosula and its therapeutic effects on non-alcoholic fatty liver disease in vitro and in vivo. *Int. J. Biol. Macromol.* 265(Pt 2)**,** 130988. doi: 10.1016/j.ijbiomac.2024.130988.

Meng, X., Kuang, H., Wang, Q., Zhang, H., Wang, D., and Kang, T. (2023). A polysaccharide from Codonopsis pilosula roots attenuates carbon tetrachloride-induced liver fibrosis via modulation of TLR4/NF-kappaB and TGF-beta1/Smad3 signaling pathway. *Int. Immunopharmacol.* 119**,** 110180. doi: 10.1016/j.intimp.2023.110180.

Qu, H., Gao, X., Cheng, C., Zhao, H., Wang, Z., and Yi, J. (2020). Hepatoprotection mechanism against alcohol-induced liver injury in vivo and structural characterization of Pinus koraiensis pine nut polysaccharide. *Int. J. Biol. Macromol.* 154**,** 1007-1021. doi: 10.1016/j.ijbiomac.2020.03.168.

Ren, F., Yang, Y., Wu, K., Zhao, T., Shi, Y., Song, M., et al. (2021). The Effects of Dandelion Polysaccharides on Iron Metabolism by Regulating Hepcidin via JAK/STAT Signaling Pathway. *Oxid. Med. Cell. Longev.* 2021**,** 7184760. doi: 10.1155/2021/7184760.

Song, J., Zhao, X., Bo, J., Lv, Z., Li, G., Chen, Y., et al. (2024). A polysaccharide from Alhagi honey protects the intestinal barrier and regulates the Nrf2/HO-1-TLR4/MAPK signaling pathway to treat alcoholic liver disease in mice. *J. Ethnopharmacol.* 321**,** 117552. doi: 10.1016/j.jep.2023.117552.

Song, X., Sun, W., Cui, W., Jia, L., and Zhang, J. (2021). A polysaccharide of PFP-1 from Pleurotus geesteranus attenuates alcoholic liver diseases via Nrf2 and NF-kappaB signaling pathways. *Food Funct.* 12(10)**,** 4591-4605. doi: 10.1039/d1fo00310k.

Sun, X., Zheng, Y., Tian, Y., Xu, Q., Liu, S., Li, H., et al. (2023). Astragalus polysaccharide alleviates alcoholic-induced hepatic fibrosis by inhibiting polymerase I and transcript release factor and the TLR4/JNK/NF-κB/MyD88 pathway. *J. Ethnopharmacol.* 314**,** 116662. doi: 10.1016/j.jep.2023.116662.

Tang, H., Zha, Z., Tan, Y., Li, Y., Jiao, Y., Yang, B., et al. (2023). Extraction and characterization of polysaccharide from fermented mycelia of Coriolus versicolor and its efficacy for treating nonalcoholic fatty liver disease. *Int. J. Biol. Macromol.* 248**,** 125951. doi: 10.1016/j.ijbiomac.2023.125951.

Teng, S., Zhang, Y., Jin, X., Zhu, Y., Li, L., Huang, X., et al. (2023). Structure and hepatoprotective activity of Usp10/NF-kappaB/Nrf2 pathway-related Morchella esculenta polysaccharide. *Carbohydr. Polym.* 303**,** 120453. doi: 10.1016/j.carbpol.2022.120453.

Wang, K., Wang, J., Song, M., Wang, H., Xia, N., and Zhang, Y. (2020). Angelica sinensis polysaccharide attenuates CCl(4)-induced liver fibrosis via the IL-22/STAT3 pathway. *Int. J. Biol. Macromol.* 162**,** 273-283. doi: 10.1016/j.ijbiomac.2020.06.166.

Wang, K., Yang, L., Zhou, J., Pan, X., He, Z., Liu, J., et al. (2022). Smilax china L. Polysaccharide Alleviates

Oxidative Stress and Protects From Acetaminophen-Induced Hepatotoxicity via Activating the Nrf2-ARE Pathway. *Front. Pharmacol.* 13**,** 888560. doi: 10.3389/fphar.2022.888560.

Wang, K., Yang, X., Wu, Z., Wang, H., Li, Q., Mei, H., et al. (2020). Dendrobium officinale Polysaccharide Protected CCl(4)-Induced Liver Fibrosis Through Intestinal Homeostasis and the LPS-TLR4-NF-kappaB Signaling Pathway. *Front. Pharmacol.* 11**,** 240. doi: 10.3389/fphar.2020.00240.

Wang, K.L., Lu, Z.M., Mao, X., Chen, L., Gong, J.S., Ren, Y., et al. (2019). Structural characterization and anti-alcoholic liver injury activity of a polysaccharide from Coriolus versicolor mycelia. *Int. J. Biol. Macromol.* 137**,** 1102-1111. doi: 10.1016/j.ijbiomac.2019.06.242.

Wang, R., Han, L., and Zhao, G.L. (2021). Protective effect andmechanism of Prunella vulgaris sulfate polysaccharide on liver injury induced by isoniazid mice. *Chin. J. Clin. Gastroenterol.* 33(4)**,** 242-245. doi: 10.3870/lcxh.j.issn.1005-541X.2021.04.04.

Wang, T., Wang, X., Zhuo, Y., Si, C., Yang, L., Meng, L., et al. (2020). Antiviral activity of a polysaccharide from Radix Isatidis (Isatis indigotica Fortune) against hepatitis B virus (HBV) in vitro via activation of JAK/STAT signal pathway. *J. Ethnopharmacol.* 257**,** 112782. doi: 10.1016/j.jep.2020.112782.

Wang, Y., Yang, J., Jin, H., Gu, D., Wang, Q., Liu, Y., et al. (2023). Comparisons of physicochemical features and hepatoprotective potentials of unprocessed and processed polysaccharides from Polygonum multiflorum Thunb. *Int. J. Biol. Macromol.* 235**,** 123901. doi: 10.1016/j.ijbiomac.2023.123901.

Yan, H., Ma, X., Mi, Z., He, Z., and Rong, P. (2022). Extracellular Polysaccharide from Rhizopus nigricans Inhibits Hepatocellular Carcinoma via miR-494-3p/TRIM36 Axis and Cyclin E Ubiquitination. *J. Clin. Transl. Hepatol.* 10(4)**,** 608-619. doi: 10.14218/JCTH.2021.00301.

Yan, T., Zhang, Y., Lu, H., Zhao, J., Wen, C., Song, S., et al. (2024). The protective effect of Enteromorpha prolifera polysaccharide on alcoholic liver injury in C57BL/6 mice. *Int. J. Biol. Macromol.* 261(Pt 2)**,** 129908. doi: 10.1016/j.ijbiomac.2024.129908.

Yan, Y., Yuan, H., Yang, F., Na, H., Yu, X., Liu, J., et al. (2024). Seabuckthorn polysaccharides mitigate hepatic steatosis by modulating the Nrf-2/HO-1 pathway and gut microbiota. *AMB Express* 14(1)**,** 100. doi: 10.1186/s13568-024-01756-7.

Yang, K., Zhan, L., Lu, T., Zhou, C., Chen, X., Dong, Y., et al. (2020). Dendrobium officinale polysaccharides protected against ethanol-induced acute liver injury in vivo and in vitro via the TLR4/NF-kappaB signaling pathway. *Cytokine* 130**,** 155058. doi: 10.1016/j.cyto.2020.155058.

Yang, Y., Liu, S., Li, H., Liu, Y., Ren, P., Liu, Y., et al. (2023). The protective effect of Nostoc commune Vauch. polysaccharide on alcohol-induced acute alcoholic liver disease and gut microbiota disturbance in mice. *J. Gastroenterol. Hepatol.* 38(12)**,** 2185-2194. doi: 10.1111/jgh.16335.

Ye, H., Ma, S., Qiu, Z., Huang, S., Deng, G., Li, Y., et al. (2022). Poria cocos polysaccharides rescue pyroptosis-driven gut vascular barrier disruption in order to alleviates non-alcoholic steatohepatitis. *J. Ethnopharmacol.* 296**,** 115457. doi: 10.1016/j.jep.2022.115457.

Yu, H., Deng, W., Chen, S., Qin, B., Yao, Y., Zhou, C., et al. (2023). Strongylocentrotus nudus egg polysaccharide (SEP) suppresses HBV replication via activation of TLR4-induced immune pathway. *Int. J. Biol. Macromol.* 245**,** 125539. doi: 10.1016/j.ijbiomac.2023.125539.

Yu, J., Liu, C., Ji, H.Y., and Liu, A.J. (2020). The caspases-dependent apoptosis of hepatoma cells induced by an acid-soluble polysaccharide from Grifola frondosa. *Int. J. Biol. Macromol.* 159**,** 364-372. doi: 10.1016/j.ijbiomac.2020.05.095.

Yu, T., He, Y., Chen, H., Lu, X., Ni, H., Ma, Y., et al. (2022). Polysaccharide from Echinacea purpurea plant ameliorates oxidative stress-induced liver injury by promoting Parkin-dependent autophagy. *Phytomedicine* 104**,** 154311. doi: 10.1016/j.phymed.2022.154311.

Zhang, L., Wang, Y., Wu, F., Wang, X., Feng, Y., and Wang, Y. (2022). MDG, an Ophiopogon japonicus polysaccharide, inhibits non-alcoholic fatty liver disease by regulating the abundance of Akkermansia muciniphila. *Int. J. Biol. Macromol.* 196, 23-34. doi: 10.1016/j.ijbiomac.2021.12.036.

Zhao, H., Li, D., Li, M., Liu, L., Deng, B., Jia, L., et al. (2022). Coprinus comatus polysaccharides ameliorated carbon tetrachloride-induced liver fibrosis through modulating inflammation and apoptosis. *Food Funct.* 13(21), 11125-11141. doi: 10.1039/d2fo01349e.

Zhao, L., Zheng, L., Li, Z., Jin, M., Wang, Q., Cheng, J., et al. (2022). Phellinus linteus polysaccharides mediates acetaminophen-induced hepatotoxicity via activating AMPK/Nrf2 signaling pathways. *Aging-US* 14, 6993-7002.

Zhao, Y., Liu, X., Ding, C., Zheng, Y., Zhu, H., Cheng, Z., et al. (2022). Aronia melanocarpa polysaccharide ameliorates liver fibrosis through TGF-beta1-mediated the activation of PI3K/AKT pathway and modulating gut microbiota. *J. Pharmacol. Sci.* 150(4), 289-300. doi: 10.1016/j.jphs.2022.10.001.

Zheng, N., Wang, H., Zhu, W., Li, Y., and Li, H. (2024). Astragalus polysaccharide attenuates nonalcoholic fatty liver disease through THDCA in high-fat diet-fed mice. *J. Ethnopharmacol.* 320, 117401. doi: 10.1016/j.jep.2023.117401.

Zhong, W., Yang, C., Zhang, Y., Liu, Y., and Yang, D. (2022). The Chemical Profiling and Anticancer Potential of Functional Polysaccharides from Flos Sophorae Immaturus. *Molecules* 27(18), 5978. doi: 10.3390/molecules27185978.
